# Amplitude response and singularity behavior of circadian clock to external stimuli

**DOI:** 10.1038/s41540-023-00300-w

**Published:** 2023-08-12

**Authors:** Tao Zhang, Yu Liu, Ling Yang

**Affiliations:** 1 Jiangsu Key Laboratory of Neuropsychiatric Diseases and Cambridge-Su Genomic Resource Center, Medical School of Soochow University, Suzhou, Jiangsu China; 2 School of Mathematical Sciences, Soochow University, Suzhou, Jiangsu China

**Keywords:** Systems biology, Computational biology and bioinformatics

## Abstract

Amplitude changes caused by environmental cues are universal in the circadian clock and associated with various diseases. Singularity behavior, characterized by the disruption of circadian rhythms due to critical stimuli, has been observed across various species. Several mathematical models of the circadian clock have replicated this phenomenon. A comprehensive understanding of the amplitude response remains elusive due to experimental limitations. In this study, we address this question by utilizing a simple normal form model that accurately fits previous experimental data, thereby presenting a general mechanism. We employ a geometric framework to illustrate the dynamics in different stimuli of light-induced transcription (LIT) and light-induced degradation (LID), highlighting the core role of invisible instability in amplitude response. Our model systematically elucidates how stimulus mode, phase, and strength determine amplitude responses. The results show that external stimuli induce alterations in both the amplitudes of individual oscillators and the synchronization among oscillators, collectively influencing the overall amplitude response. While experimental methods impose constraints resulting in limited outcomes under specific conditions, our model provides a comprehensive and three-dimensional mechanistic explanation. A comparison with existing experimental findings demonstrates the consistency of our proposed mechanism. Considering the response direction, the framework enables the identification of phases that lead to increased circadian amplitude. Based on this mechanism derived from the framework, stimulus strategies for resetting circadian rhythms with reduced side effects could be designed. Our results demonstrate that the framework has great potential for understanding and applying stimulus responses in the circadian clock and other limit cycle oscillations.

## Introduction

A biological timer called circadian clock is generated in virtually all organisms to align physiology and behavior with the daily light–dark cycle and adapt to diurnal environmental changes^1^. Such circadian rhythms are endogenous and self-sustained, persisting with a period of ~24 hours even in the absence of external signals^2^. In mammals, the central pacemaker resides in the hypothalamic suprachiasmatic nucleus (SCN), which is composed of ~20,000 coupled autonomous oscillators. The SCN receives photic signals from the retina and coordinates central and peripheral clocks^3^. Additionally, environmental cues gate the molecular clock by regulating transcription and protein synthesis of core clock components, allowing organisms to adjust to changes in their surroundings^4–6^.

Environmental cues, such as light, can reset the biological clock by causing phase shifts and amplitude changes. The clock exhibits phase-specific responses to resetting signals, as demonstrated by phase response curves (PRCs) and amplitude response curves (ARCs)^7^. Previous studies on circadian responses have primarily focused on phase shifts, and extensive documentation exists at the molecular, cellular, and behavioral levels^8,9^. However, it is crucial to recognize the significance of amplitude response in the clock system, as an enhanced amplitude can combat metabolic disease and cancer^10,11^. Disruptions in circadian rhythms characterized by attenuated amplitudes have been implicated in many pathological processes, such as depressive disorder, diabetes, cardiovascular disease, and cancer. Furthermore, it is also noteworthy that amplitude change and phase shift often occur concurrently during stimulation, such as during jetlag, which is accompanied by pronounced amplitude reduction^9,12^. These observations underscore the importance of amplitude regulation in maintaining physiological homeostasis^13–15^. However, the amplitude response has received comparatively less attention in research, primarily due to the difficulty associated with experimental measurement.

Singularity behavior, characterized by the extreme suppression of circadian rhythms by critical perturbation, is the most dramatic phenomenon in the amplitude response area. Singularity behavior has been found in various species, including Neurospora, Drosophila, hamsters, and humans, which are triggered at different phases in different species^16–19^. Alternative mechanisms are suggested from in vitro, in vivo, and in silico studies to explain singularity behavior, including (1) arrhythmicity in all oscillators, (2) desynchronization of the population, or both^7,17,20,21^. Nevertheless, ongoing debates persist regarding the precise underlying mechanism governing the amplitude response to distinct external stimuli.

Many mathematical models have been proposed to investigate the resetting of the circadian clock^21–28^. These models include complex molecular models, simplified normal form models, and geometric frameworks, which have successfully mimicked experimental results. These models are typically constructed using one or more ordinary differential equations (ODEs) to capture the behavior and dynamics of a system over time^7,17,29^. However, due to the large number of variables and parameters, it is virtually impossible that all variables and parameters are accurate. The accuracy of the ODE models is limited to specific circumstances where the assumptions hold. Geometric frameworks are commonly employed to study changes in external behavior because they do not contain complete internal information^20^. The normal form model, being the most elementary dynamic model, is capable of elucidating the stimulus-response mechanism, despite its simplicity in representing fundamental regulatory processes. As a result, different types of models are employed to address different research questions and tackle specific problems in circadian clock studies.

We employed a normal form model and a detailed model to simulate the response of the circadian amplitude to external light stimuli. Furthermore, we compared the model results with the experimental findings of previous studies on the light-induced response of the circadian clock amplitude in NIH 3T3 cells^7^. Based on the simulation results, we found that the normal form model agrees better with the experimental results than the detailed model. In this study, we utilized the normal form model to illustrate the amplitude response. The results show that the stimulus mode, phase, and strength determine the amplitude response. Moreover, synchronization of the cell population and single-cell amplitude contribute to average amplitude changes collaboratively. The amplitude response mechanism can be derived using a geometric framework. Additionally, we design a combined stimulus strategy within the framework to reset the circadian phase without reducing amplitude. Furthermore, in most biological systems, stability plays a vital role because it is easy to observe, such as the stable limit cycles in the circadian clock, cell cycle, and glycolytic oscillations^30–34^. However, our study suggests that instability plays a crucial role in the amplitude response and that the principle governing amplitude regulation is highly dependent upon it.

## Results

### Amplitude response to light pulse: experimental observations and numerical simulations

To simulate and analyze the amplitude responses of circadian clocks to external stimuli, we used light pulses to induce amplitude responses and summarized them into general rules. In various species, light exposure triggers transcriptional activation and protein degradation processes. To incorporate these processes into models, we introduced modifications to both a normal form model and a detailed model, including (1) adding an external stimulus module to the model, to simulate phenomena such as transcriptional activation induced by light, and (2) adding intercellular coupling to simulate the oscillations of cell populations and their amplitude response to external stimuli (see details in methods)^29,35^. All modified models demonstrated stable oscillations in the absence of external stimuli (Supplementary Fig. 1). To quantify the extent of the amplitude response after stimulation, we defined four indices (see details in methods). The amplitude response of a single oscillator to a light pulse was represented by *R*_A_, which denotes the ratio of amplitudes in the 24 hours before and after stimulation. *R*_AA_ represents the ratio of the average amplitude before and after stimulation, characterizing the amplitude response of the population. *R*_IA_ and *R*_IS_ are used to indicate the amplitude changes within individual cells and changes in intercellular synchronization, respectively.

**Fig. 1:**
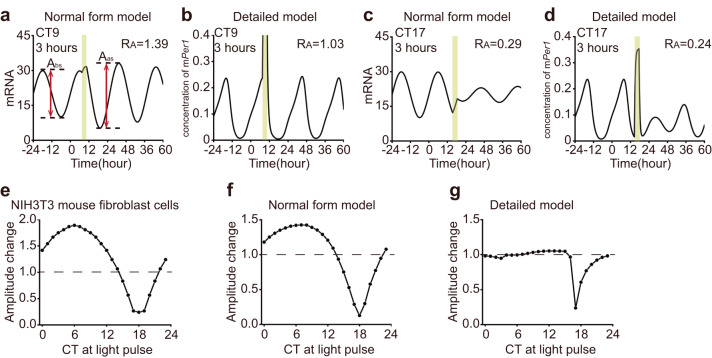
Amplitude response from the model of a single oscillator and experimental data. **a**–**d** Numerical simulation results of mRNA concentration from the normal form (**a**, **c**) and detailed (**b**, **d**) model of a single oscillator. The timing of the light pulse (yellow bar) is designated in each panel. The stimulus condition is labeled in the upper left, and the amplitude response is labeled in the upper right. The amplitudes before and after stimulation are indicated by red double arrow lines (**a**). **e**, **g** The amplitude response curves (ARCs) of NIH3T3 mouse fibroblast cells (**e**), as well as those of the normal form (**f**) and detailed models (**g**), are plotted against the timing of light pulses in CT.

To investigate the mechanisms underlying the amplitude response, we employed both the normal form model and the detailed model, each consisting of a single oscillator, to simulate the amplitude response to light-induced transcriptional activation (LIT). For instance, upon applying a 3-hour LIT at CT9, both the normal form model and the detailed model exhibited an increase in amplitude, which qualitatively matched experimental findings^7^. However, the amplitude increase observed in the detailed model was relatively small (Fig. 1a, b, *R*_A_ = 1.39 and 1.03). Conversely, when a 3-hour LIT was applied at CT17, a critical perturbation known to trigger singularity behavior^7^, both models reproduced a heavily suppressed oscillating amplitude (Fig. 1c, d, *R*_A_ = 0.29 and 0.24). To compare the simulated amplitude response with that of the experiment, we obtained the experimental amplitude responses to a 3-hour light pulse in NIH3T3 mouse fibroblast cells from a previous study and plotted corresponding ARCs (Fig. 1e)^7^. Additionally, we measured the amplitude responses to a 3-hour LIT at each circadian time using both the normal form model and the detailed model (Fig. 1f, g). Qualitatively, amplitude responses generated by the normal form model exhibited closer similarity to the experimental results compared to those produced by the detailed model.

**Fig. 2:**
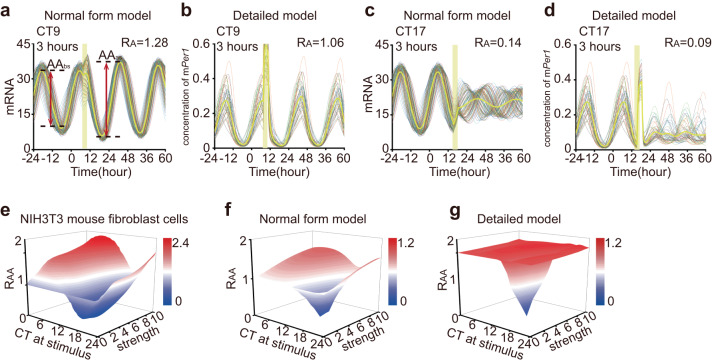
Amplitude response from the model of coupled oscillators and experimental data. **a**–**d** Numerical simulation results of mRNA concentration from the normal form (**a**, **c**) and detailed (**b**, **d**) model of 100 coupled oscillators. The timing of the light pulse (yellow bar) is designated in each panel. The stimulus condition is labeled in the upper left, and the average amplitude response is labeled in the upper right. The yellow curve represents the average output. The amplitudes before and after stimulation are indicated by red double arrow lines (**a**). **e**–**g** Average amplitude response surfaces of NIH3T3 mouse fibroblast cells (**e**), as well as those of the normal form (**f**) and detailed models (**g**), which are 3D plots of average amplitude response vs. stimulus timing and strength. Red represents the strongest; blue represents the weakest.

In mammals, sustained circadian oscillation arises from robust individual rhythm and intercellular communication. External perturbation simultaneously altered the amplitude of individual cells and synchronization among them. Therefore, we incorporated couplings into both models to simulate the amplitude responses of the cell population to LIT. Consistent with the experimental results and single-cell models, a 3-hour LIT at CT9 increased the amplitude of the modeling cell population (Fig. 2a, b, *R*_AA_ = 1.28 and 1.06). Moreover, individual amplitude and intercellular coupling were enhanced by a 3-hour LIT at CT9 (Fig. 2a, b, *R*_IA_ = 1.17 and 1.07, *R*_IS_ = 1.08 and 1.05). Conversely, when we delivered a 3-hour LIT at CT17, a decrease in single-cell amplitude and complete desynchronization were observed (Fig. 2c, d, *R*_IA_ = 0.43 and 0.45, *R*_IS_ = 0.40 and 0.43). The average output of cell population completely stopped oscillating during the first day after the stimulus and maintained a very low amplitude over the subsequent two days (Fig. 2c, d, *R*_AA_ = 0.14 and 0.09).

To obtain a systematic understanding of the amplitude response and facilitate a comparison between simulation and experimental results, we extracted the experimental data on amplitude responses to light pulses in NIH3T3 mouse fibroblast cells from a previous study and visualized them as an amplitude response surface (ARS) (Fig. 2e)^7^. Additionally, using a normal form model and a detailed model, we simulated the amplitude responses to LIT at different timings and with varying strengths, which were then represented as the ARS (Fig. 2f, g). Although the detailed model is more intricate than the normal form model, the amplitude responses obtained from the normal form model resemble the experimental data more closely.

Therefore, the amplitude response is a pervasive phenomenon observed both in experiments and through numerical simulation. The qualitative agreement between the experimental observations and the results obtained from the two models further supports this notion. Moreover, from the perspective of amplitude response, the normal form model emerges as the suitable choice. Consequently, we employ the simplified model to elucidate the underlying mechanisms involved in amplitude responses.

### The geometric framework

Under constant environmental conditions, circadian rhythms are characterized as states of clock cycling along a closed trajectory known as a ‘limit cycle’’. To intuitively demonstrate the process of amplitude response, the system of the normal form model is transformed into polar coordinate form

1 $$\frac{d{\theta }}{{dt}}=\omega$$

2 $$\frac{{dr}}{{dt}}=\epsilon r({a}^{2}-{r}^{2})$$

It is obvious that system (Eqs. (1) and (2)) has the following three distinct properties:

1. A circular limit cycle with radius *a* exists (Fig. 3a, red line),

**Fig. 3:**
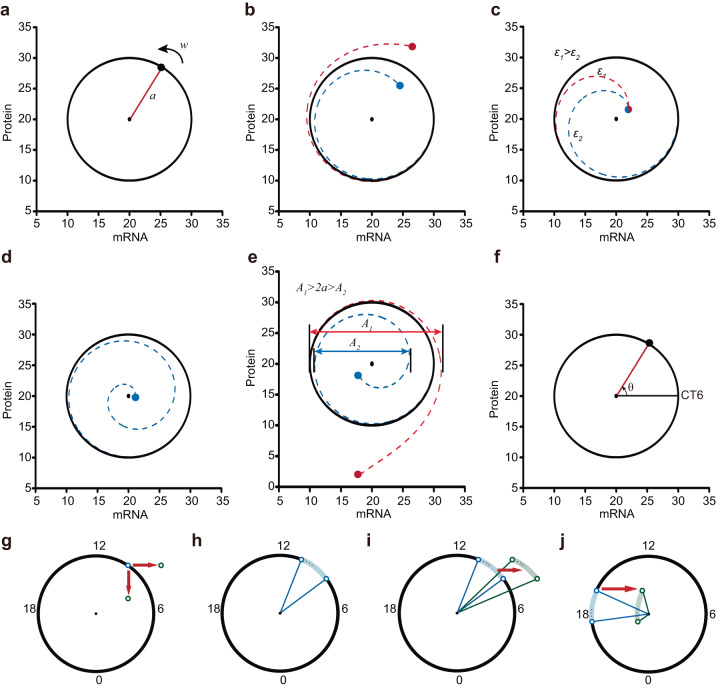
Geometric framework for interpreting amplitude changes. **a**–**f** Dynamic properties of the normal form system are shown in polar coordinates. The radius of the circular limit cycle is *a*, and the constant velocity of each trajectory is *ω* (**a**). Trajectories are attracted to the limit cycle (**b**), and the parameter *ε* determines the rate (**c**). States near the unstable point run around the point and toward the stable limit cycle (**d**). The difference between the maximum and minimum mRNA levels within one cycle in the trajectory is the amplitude (double arrows) (**e**). The phase corresponding to the angle of 0 degrees (positive direction) is that of CT6 (**f**). **g**, **h** The geometric framework illustrates stable circadian oscillations as a two-dimensional (mRNA and protein) circular limit cycle. **g** Blue and green hollow circles represent oscillators before and after stimulation. CT18 is the time point when the mRNA reaches its trough. The red arrow represents the stimulus. **h** The blue curved shadow demonstrates the oscillators before stimulation. The two blue lines represent the amplitude of the two oscillators before stimulation on the boundary. The angle between two boundary lines represents the synchronization level of the oscillators. **i**, **j** Mechanism interpretation of the amplitude response in Fig. 1a–d in the geometric framework.
2. The angular velocity of each trajectory is constant at *ω* (Fig. 3a),
3. Each trajectory converges toward the limit cycle (Fig. 3b), and the rate of convergence is determined by *ε* (Fig. 3c).

The system possesses an unstable point (the center). This unstable steady state has been acknowledged and utilized in various biological studies, particularly in clock resetting^17,36^. States near the unstable point revolve around it and move away from it (Fig. 3d). Although the unstable point is not as readily identifiable as the stable steady points or the stable limit cycles, it plays a critical role in the amplitude response. This point is referred to as the singularity point^37^, and all the trajectories revolve around it.

In the state-space representation, the amplitude of the trajectory is defined as the difference between the maximum and minimum values of mRNA within one cycle, illustrated by double arrows (Fig. 3e). If the initial state lies outside the limit cycle, the amplitude increases (Fig. 3e, red double arrows), whereas if it lies within the limit cycle, the amplitude decreases (Fig. 3e, blue double arrows). In the case of stable oscillations, the amplitude is 2*a*. The angle between the positive direction of *x* axis (mRNA) and the radial line corresponds to the circadian phase. According to the experimental results, the phase corresponding to an angle of 0 degrees (in the positive direction) is CT6 (Fig. 3f)^7^.

Therefore, based on the aforementioned characteristics, we can geometrically illustrate the trajectory after perturbation using a demonstration system called the geometric framework. This framework facilitates the intuitive understanding of changes in cellular synchronization and individual amplitudes. In this framework, external stimulation can push the oscillator away from the limit cycle, altering its phase and the radial distance between the oscillator and the singularity point. Once the stimulation is removed, the oscillator gradually returns to the limit cycle. LIT is depicted as rightward red arrows, while LID is represented by downward red arrows (Fig. 3g). In an organism, the states of all cells are not identical, as there are slight variations due to interindividual differences and environmental noise. The dots within the curved shadow and two hollow circles mimic a population of approximately synchronized cells (Fig. 3h). The level of synchronization among oscillators is visually indicated by the angle between two boundary lines (Fig. 3h, angle between blue lines). Oscillators after the light pulse are exemplified by dots within the curved green shadow and two hollow green circles (Fig. 3i).

Next, we aimed to provide an explanation for the simulation results depicted in Fig. 2a–d using the geometric framework. At CT9, a 3-hour LIT (rightward red arrows) drove the oscillators away from the stable limit cycle, resulting in higher single-cell amplitudes and smaller phase differences (Fig. 3i). Conversely, when a 3-hour LIT stimulus was applied at CT17, the oscillators were near the singularity point, leading to significant suppression of amplitude and desynchronization of phases among the oscillators (Fig. 3j).

In summary, under normal conditions, oscillators exhibit cyclic behavior along the limit cycle, while external stimulation can disrupt this closed trajectory and perturb the phases of oscillators, leading to changes in intercellular synchronization. However, once the stimulation is removed, the oscillators tend to return to the stable limit cycle. The amplitude changes can be observed by analyzing the mean trajectory of the oscillators after completing a cycle following the stimulation. The findings obtained from the geometric framework align well with the results of the normal form model, and the framework provides a concise and intuitive way to illustrate and explain both intercellular synchronization and intracellular rhythm responses. These schematic interpretations demonstrate that the geometric framework serves as a valuable tool for understanding and elucidating the mechanisms underlying amplitude responses in circadian systems.

### Singularity behavior and its mechanism

The ARS serves as a valuable tool for quantifying the extent of global amplitude changes (Fig. 2e–g). One notable characteristic of this surface is the presence of a distinct minimum point, which signifies the lowest amplitude response and indicates a significant reduction in average amplitude. Termed singularity behavior, this phenomenon arises as a result of exposure to a critical stimulus. Singularity behaviors have been observed in a range of organisms, including cyanobacteria, *Neurospora crassa*, Drosophila, hamsters, and humans^17–20^. However, the underlying mechanism responsible for singularity behavior remains a subject of ongoing controversy and debate.

In the simplified normal form model, singularity behavior occurred at CT17 with a 3-hour LIT (*R*_AA, LIT_ = 0.14), which is consistent with the experimental findings (Fig. 2c)^7^. Singularity behavior has been previously attributed to either amplitude suppression within individual cellular clocks or desynchronization among cellular clocks^7,17^. Figure 2c demonstrates a significant reduction in amplitudes across all cells and a pronounced desynchronization of phases at CT17 (*R*_IA, LIT_ = 0.43, *R*_IS, LIT_ = 0.40).

**Fig. 4:**
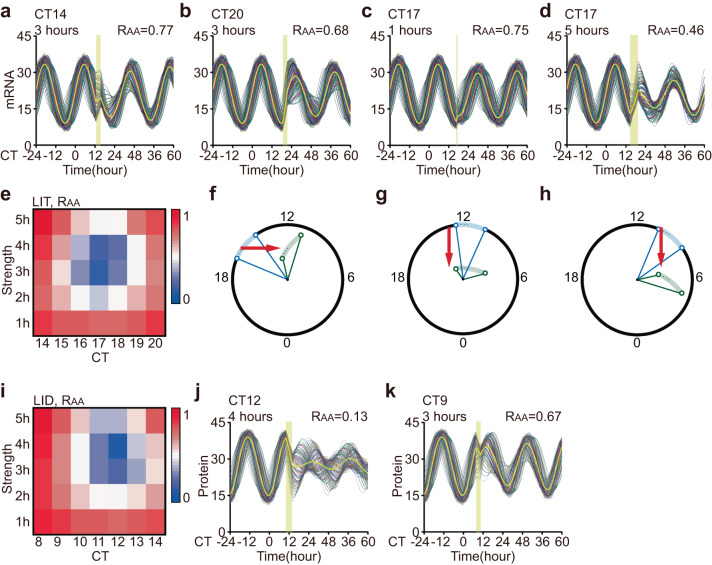
Singularity behavior and mechanism. **a**–**d** The response to LIT of 100 coupled oscillators and their averages (yellow lines) are shown in four different cases. The timing of the light pulse (yellow bar) is designated in each panel. The stimulus condition is labeled in the upper left, and the average amplitude response is labeled in the upper right. The yellow curve represents the average output. **e** Heatmap of the average amplitude response to LIT. The *R*_AA_ value (shown in the color bar) is plotted against stimulus timing (CT14 to CT20) and strength (1 hours to 5 hours). **f** Mechanism interpretation of nonsingular behavior by LIT (CT14, 3 hours) in the geometric framework. **g**, **h** Mechanistic interpretation of singularity behavior by LID in a geometric framework. **i** Heatmap of the average amplitude response to LID. The *R*_AA_ value (shown in the color bar) is plotted against stimulus timing (CT8 to CT14) and strength (1 h–5 h). **j**, **k** The response to LID of 100 coupled oscillators and their averages (yellow lines) are shown in two different cases. The timing of the light pulse (yellow bar) is designated in each panel. The stimulus condition is labeled in the upper left, and the average amplitude response is labeled in the upper right. The yellow curve represents the average output.

Singularity behavior relies on both stimulus phase and strength. We examined the amplitude response to light pulses at different CTs and with varying durations. At CT14 or CT20, a 3-hour LIT stimulus did not lead to a significant reduction in average amplitudes (*R*_AA, LIT_ = 0.77 and 0.68) (Fig. 4a, b). However, single-cell amplitudes were decreased (*R*_IA, LIT_ = 0.81 and 0.76) while maintaining synchronization among cells (*R*_IS, LIT_ = 0.95 and 1.00) after the light pulse. Similarly, a 1-hour or 5-hour LIT at CT17 resulted in attenuated average amplitudes (*R*_AA, LIT_ = 0.75 and 0.46), but singularity behavior was not observed (Fig. 4c, d). In these cases, individual cellular rhythms became weaker (*R*_IA, LIT_ = 0.84 and 0.55) while maintaining a relatively high level of synchrony among cells (*R*_IS, LIT_ = 0.93 and 1.05). We then converted the three-dimensional surface into a heatmap to identify the stimulus combinations that induced singularity behavior (Fig. 4e). The heatmap revealed that LIT triggered singularity behavior in a narrow time window around CT17, with a duration of approximately 3 hours. Therefore, singularity behavior is contingent upon specific stimulus timing and strength, consistent with previous studies^10,26^. Furthermore, amplitude loss and desynchronization emerged collectively during amplitude suppression.

To provide an intuitive illustration of the mechanism underlying singularity behavior, we sought to illustrate responses to a critical stimulus using the geometric framework. When compared with the results shown in Fig. 3j, a 3-hour LIT at other phases (e.g., CT14) does not cause such low single-cell amplitudes and large phase differences as singularity stimulation (Fig. 4f). Considering the mechanism of singularity behavior by LIT, we hypothesized that LID could trigger singularity around CT12 with an appropriate strength, while at other phases (e.g., CT9), the amplitude would only decrease moderately (Fig. 4g, h). Therefore, we performed a scan within the range of the stimulus phase from CT8 to CT14 and strength from 1 to 5 hours (Fig. 4i). The results show that singularity behavior emerged from a 4-hour LID at CT12 instead of the other phases (e.g., CT9) (Fig. 4j, k*R*_AA, LID_ = 0.13, *R*_AA, LID_ = 0.67). Similar to the singularity induced by LIT, both the suppression of single-cell amplitudes and desynchronization contribute to a severely suppressed average amplitude after the 4-hour LID at CT12 (*R*_IA, LIT_ = 0.37, *R*_IS, LIT_ = 0.13).

These simulation results and the corresponding interpretation within the geometric framework confirm that the two alternative explanations for singularity behavior are not exclusive. The specific stimulus, whether it is LIT or LID, pushes oscillators close to the singularity point, resulting in both separated phases and weakened rhythms of individual clocks. Furthermore, the critical phase at which singularity behavior occurs is determined by the specific mode of the stimulus. Typically, research tends to focus on stable steady states that are observable and measurable. While the unstable steady state is inherently invisible, they play a pivotal role in singularity phenomena.

Singularity behaviors are harmful to organisms due to the extreme weakening of circadian rhythms. Robust circadian rhythms play an important role in maintaining homeostasis. Thus, an important question is raised: what are the stimulus conditions that can improve the circadian amplitude?

### Monotonic increasing average amplitude responses

**Fig. 5:**
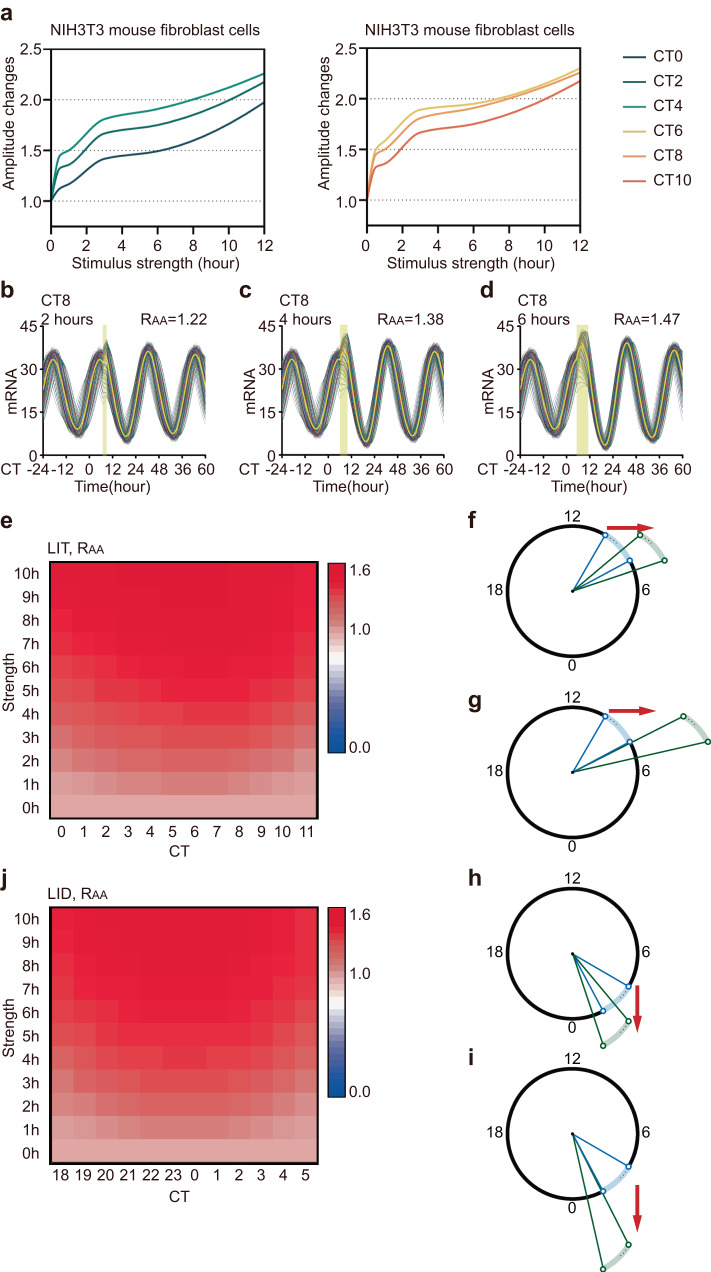
Monotonic increasing average amplitude responses. **a** The amplitude responses of NIH3T3 mouse fibroblast cells from CT0 to CT10 are plotted against the stimulus strength. **b**–**d** The response to LIT of 100 coupled oscillators and their averages (yellow lines) are shown in three different cases. The timing of the light pulse (yellow bar) is designated in each panel. The stimulus condition is labeled in the upper left, and the average amplitude response is labeled in the upper right. The yellow curve represents the average output. **e** Heatmap of the average amplitude response to LIT. The *R*_AA_ value (shown in the color bar) is plotted against stimulus timing (CT0 to CT11) and strength (0 h to 10 h). **f**, **g** Mechanistic interpretation of the monotonous average amplitude response by LIT in the geometric framework. **h**, **i** Mechanistic interpretation of the monotonous average amplitude response by LID in the geometric framework. **j** Heatmap of the average amplitude response to LID. The *R*_AA_ value (shown in the color bar) is plotted against stimulus timing (CT18 to CT5) and strength (0 h to 10 h).

To elucidate the stimulus phase associated with amplitude enhancement, we analyzed the experimental data on amplitude responses to light pulses in NIH3T3 mouse fibroblast cells obtained from a previous study^7^. Our findings revealed that when stimuli were applied between CT0 and CT11, the amplitude response exhibited a monotonically increasing trend with stimulus strength, consistently exceeding 1 (Fig. 5a). Subsequently, we employed the normal form model to simulate the amplitude response from CT0 to CT11. Specifically, when the light pulse was applied at CT8, we observed that the average amplitude of the cell population was enhanced in response to increasing stimulus strength by LIT (Fig. 5b–d). To provide a comprehensive representation of average amplitude responses induced by stimuli across CT0 to CT11, we generated a heatmap-based simulated amplitude response (Fig. 5e). Remarkably, we observed that LIT effectively enhanced the average amplitude as the stimulus strength increased (Fig. 5e). Additionally, LIT demonstrated the capability to elevate single-cell amplitude and promote synchronization among cellular clocks regardless of the stimulus strength from CT0 to CT11 (Supplementary Fig. 2a, b). Additionally, both single-cell amplitude and intercellular synchronization were enhanced by increasing stimulus strength (Supplementary Fig. 2a, b).

Next, we visually represented these responses using the geometric framework. When the light pulse was applied around CT8, oscillators were displaced outward from the stable limit cycle. As a result, the single-cell amplitudes increased, and the phase differences decreased compared to the prelight pulse state (Fig. 5f). Stronger stimulation pushed the oscillators farther away from the limit cycle, resulting in greater enhancement of the single-cell amplitude and intercellular coupling (Fig. 5g). This illustration implies that when the light pulse induces protein degradation rather than transcription, the corresponding phase where the average amplitude response monotonically increases with stimulus strength is 6 hours ahead for LIT (CT18 to CT5) (Fig. 5h, i). To validate this hypothesis, we measured the amplitude changes in proteins following LID from CT18 to CT5 (Fig. 5j). The results clearly demonstrate an increasing trend in the average amplitude with rising stimulus strength, with all *R*_AA_ values exceeding 1 (Fig. 5j). Additionally, both *R*_IA_ and *R*_IS_ increased with stimulus strength and were greater than one from CT18 to CT5 (Supplementary Fig. 2c, d).

Based on the above results, when stimuli are applied at phases where oscillators can be pushed away from the singularity, there is a consistent enhancement of the average amplitude. These phases, which facilitate an increase in amplitude, can be identified within the geometric framework.

### Nonmonotonic average amplitude responses

**Fig. 6:**
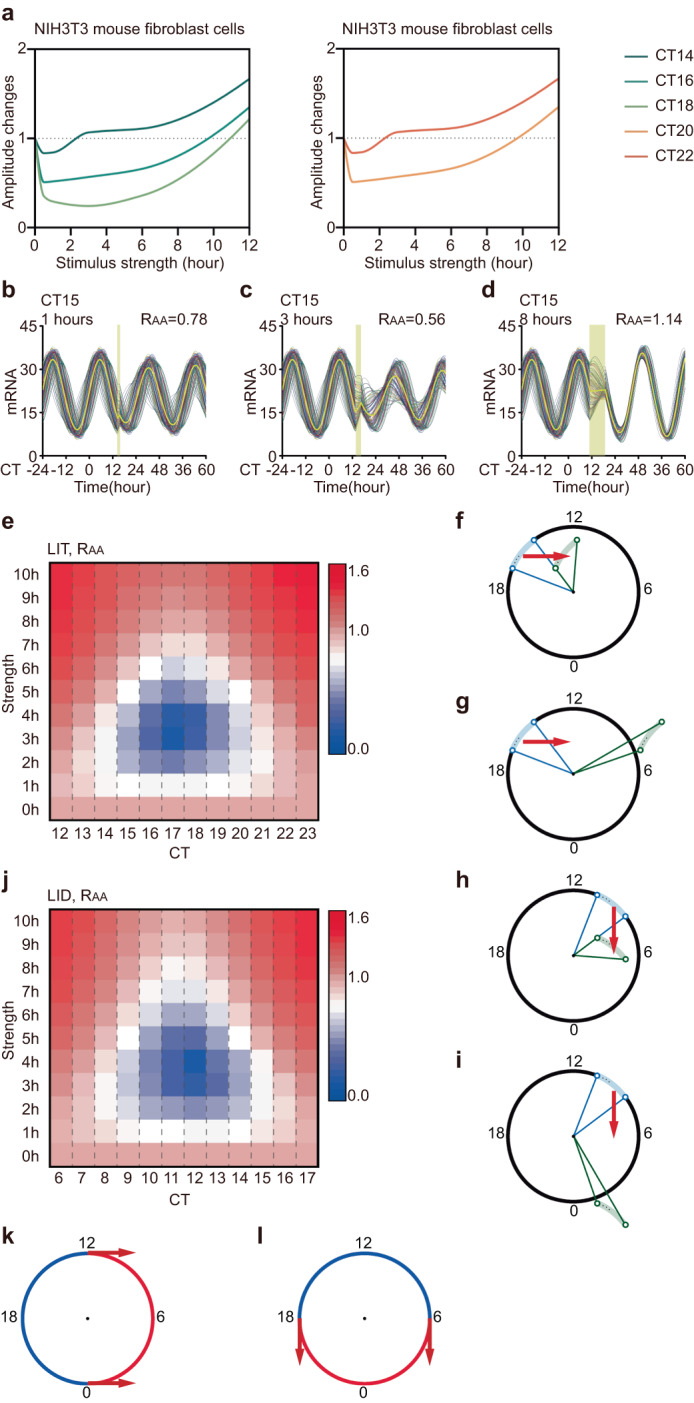
Nonmonotonic average amplitude responses. **a** The amplitude responses of NIH3T3 mouse fibroblast cells from CT14 to CT22 are plotted against the stimulus strength. **b**–**d** The response to LIT of 100 coupled oscillators and their averages (yellow lines) are shown in three different cases. The timing of the light pulse (yellow bar) is designated in each panel. The stimulus condition is labeled in the upper left, and the average amplitude response is labeled in the upper right. The yellow curve represents the average output. **e** Heatmap of the average amplitude response to LIT. The *R*_AA_ value (shown in the color bar) is plotted against stimulus timing (CT12 to CT23) and strength (0 h to 10 h). **f**, **g** Mechanistic interpretation of the nonmonotonic average amplitude response by LIT in a geometric framework. **h**, **i** Mechanistic interpretation of the nonmonotonic average amplitude response by LID in the geometric framework. **j** Heatmap of the average amplitude response to LID. The *R*_AA_ value (shown in the color bar) is plotted against stimulus timing (CT6 to CT17) and strength (0 h to 10 h). **k**, **l** Diagram illustration of the phases of nonmonotonic (blue) and monotonic (red) average amplitude response by LIT (**k**) and LID (**l**) in the geometric framework.

LIT induces a monotonically increasing amplitude response from CT0 to CT12. However, we were curious whether light pulses delivered in the other phase range (CT13 to CT23) could result in either monotonic or erratic average amplitude changes. Additionally, singularity behavior is a specific phenomenon that occurs near the circadian nadir at CT17. Therefore, we aimed to investigate the amplitude response following the administration of stimuli with varying strengths during these phases (CT13 to CT23). Experimental data from NIH3T3 mouse fibroblast cells showed that the average amplitude response to light exhibited an initial decrease followed by an increase as stimulus strength increased (Fig. 6a). Then, we utilized the normal form model to simulate the amplitude response during CT12 to CT23. Figure 6b shows that 1-hour LIT at CT15 caused a diminished average amplitude (*R*_AA_ = 0.78). Moreover, Fig. 6c illustrates that a 3-hour LIT further attenuated the average amplitude at CT15 (*R*_AA_ = 0.56). However, amplitude responses were increased when an 8-hour LIT was applied at CT15 (*R*_AA_ = 1.14) (Fig. 6d). To reach the general conclusion, we plotted the responses during the circadian trough of mRNA as a function of stimulus strength and phase (Fig. 6e). The heatmap demonstrates that at each phase, the average amplitude responses to LIT initially decreased and then increased with increasing stimulus strength (Fig. 6e). Moreover, similar trends were observed in the responses of single-cell amplitudes and intercellular coupling. The minimum response (e.g., singularity behavior) was encountered at a specific stimulus strength (3 hours or 4 hours) and then increased (Fig. 6e, Supplementary Fig. 3a, b).

Subsequently, we visually depicted the responses occurring during the circadian trough using the geometric framework in an intuitive manner. At CT15, a weak LIT pushes the oscillators close to the singularity, which leads to low amplitudes of individual cells and large phase differences (Fig. 6f). A stronger LIT can bring the oscillators outside the limit cycle, resulting in higher cellular amplitudes and smaller phase differences (Fig. 6g). Based on the mechanism illustrated in the framework, we hypothesized that LID could induce similar nonmonotonic responses during CT6 to CT17, when the protein level is higher than the average (Fig. 6h, i). Then, we measured average amplitude responses to LID from CT6 to CT17 with different strengths and found a trend very similar to that observed in LIT (Fig. 6j). Weak stimulation dampened single-cell amplitudes and spread phases, while strong stimulation enhanced single-cell amplitudes and intercellular coupling (Supplementary Fig, 3c, d).

Considering these results, it is evident that stimuli applied at phases where oscillators can be pushed close to the singularity do not always weaken the average amplitude like a singularity stimulus. In fact, strong stimulation can improve the average amplitude even at a specific phase associated with singularity behavior. The identification of phases where stimuli can elicit these nonmonotonic responses can be achieved by using the geometric framework.

Furthermore, according to the interpretations and predictions derived from the schematic diagram within the framework demonstration, specific phases of certain amplitude responses are determined by the direction of the response (Fig. 6k, l). As shown in Fig. 6k, l, phases represented by blue and red semicircles correspond to nonmonotonically changing and monotonically increasing average amplitude responses, respectively. Notably, there is a 6-hour shift in phase range resulting from different types of stimulation (Fig. 6e, j–l).

**Fig. 7:**
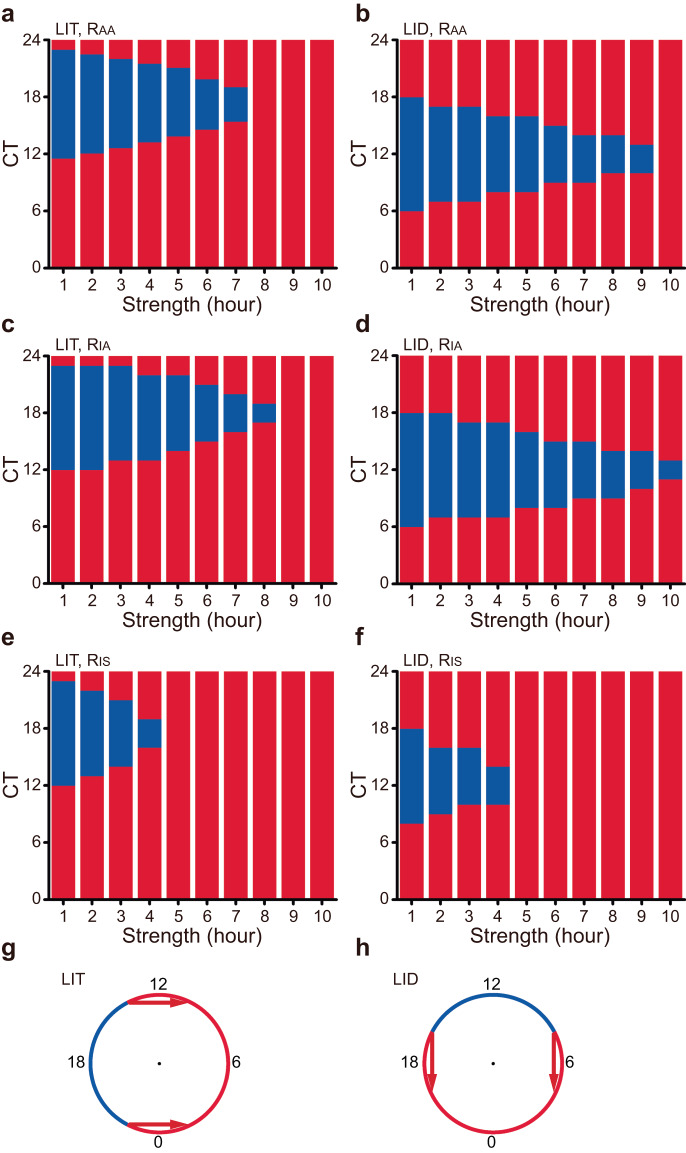
Range of amplitude reduction was less than 12 hours. **a**, **b** Stacked columns for average amplitude response to LIT (**a**) and LID (**b**), red: *R*_AA_ > 1, blue: *R*_AA_ < 1. **c**, **d** Stacked columns for single-cell amplitude response to LIT (**c**) and LID (**d**), red: *R*_IA_ > 1, blue: *R*_IA_ < 1. **e**, **f** Stacked columns for population synchronization in response to LIT (**e**) and LID (**f**), red: *R*_IS_ > 1, blue: *R*_IS_ < 1. **g**, **h** Diagram illustration of the phases of *R*_AA_ < 1 (blue) and *R*_AA_ > 1 (red) by LIT (**g**) and LID (**h**) in the geometric framework.

Finally, we calculated the range of amplitude responses that were less than one under different stimulus strengths. We found that the phase range with *R*_AA_ < 1 (blue block) decreased as the stimulus strength increased, regardless of LIT or LID (Fig. 7a, b). Even under the weakest stimulation (the first bar), the range of amplitude reduction was less than 12 hours. Furthermore, the phase range associated with a single-cell amplitude decrease and desynchronization was less than 12 hours (Fig. 7c–f). The phase ranges with *R*_AA_ < 1, *R*_IA_ < 1, and *R*_IS_ < 1 exhibited a 6-hour shift between LIT and LID (Fig. 7a–f). This shift in phase ranges can be attributed to the different response directions of LIT and LID. The two critical phases where the amplitude remained unchanged can be visually represented within the geometric framework (Fig. 7g, h).

These findings also suggest that over half of the combinations of timing and strength have the potential to increase the average amplitude. The phases associated with specific amplitude responses can be estimated by considering the response direction within the framework. This indicates that by understanding the response direction and considering the appropriate timing and strength of the stimulus, it is possible to enhance the average amplitude in circadian rhythms. The underlying principle behind these strategies is that the amplitude response depends on the relative positioning of the post-stimulus state and the unstable steady state.

### Combined stimulus strategy to regulate amplitude

We have interpreted the mechanisms underlying amplitude responses to LIT and LID using a geometric framework. However, importantly, the amplitude change is limited to that of a single stimulus due to response saturation. Moreover, it is crucial to acknowledge that modifying either the amplitude or the phase through a single stimulus can potentially result in a decrease in amplitude or a phase shift. Consequently, to achieve the desired response without any undesirable side effects, we design combined stimulus strategies within the geometric framework.

**Fig. 8:**
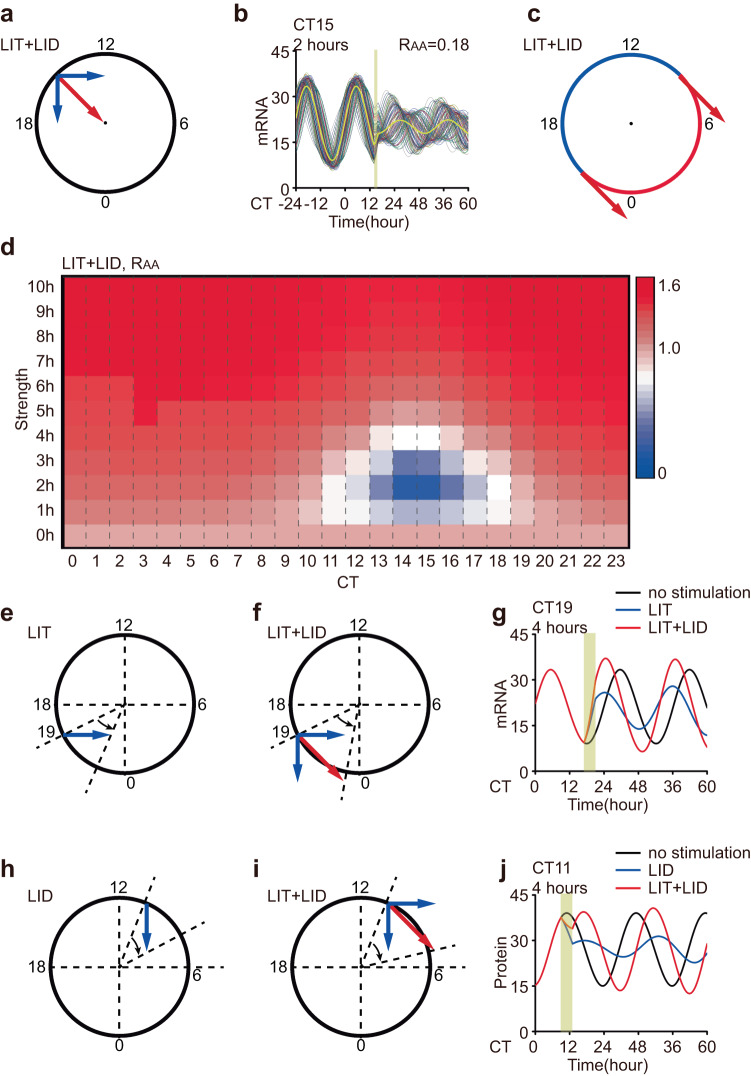
Combined stimulus strategy in the geometric framework. **a** An illustration of the phase of singular behavior by LIT and LID together in a geometric framework. **b** The response to combined stimulus of 100 coupled oscillators and their averages (yellow lines). The timing of the light pulse (yellow bar) is designated. The stimulus condition is labeled in the upper left, and the average amplitude response is labeled in the upper right. **c** Diagram illustration of the phases of nonmonotonic (blue) and monotonic (red) amplitude responses by combined stimulus in the geometric framework. **d** Heatmap of average amplitude response to combined stimulus. The *R*_AA_ value (shown in the color bar) is plotted against stimulus timing (CT0 to CT23) and strength (0 h to 10 h). **e** Framework illustration that a single LIT stimulus advances the phase but reduces the amplitude (blue arrow) at CT19. **f** Framework illustration of a combined stimulus (red arrow) that can lead to approximately the same phase advance without amplitude reduction by a single stimulus at CT19. **g** The average response to a single LIT and combined stimulus of 100 coupled oscillators at CT19. The timing of the light pulse (yellow bar) is designated in each panel. **h** Framework illustration of a single LID stimulus that delays the phase but reduces the amplitude (blue arrow) at CT11. **i** Framework illustration of a combined stimulus (red arrow) that can lead to approximately the same phase delay without amplitude reduction by a single stimulus at CT11. **j** The average response to a single LID and combined stimulus of 100 coupled oscillators at CT11. The timing of the light pulse (yellow bar) is designated in each panel.

To achieve specific goals, it may be necessary to induce singular behavior in the circadian rhythm of organisms^18^. Previous analyses have shown that strong LIT brings the oscillators closer to singularity points, resulting in the suppression of the circadian rhythm. Nevertheless, if the system is already heavily saturated in terms of stimulus-response, no matter how strong the stimulus is, it cannot be pushed closer to the singularity point. To induce singularity behavior, we simultaneously administered weak LIT and LID at CT15 (Fig. 8a). The simulation results show that the 2-hour LIT and LID caused a weakened amplitude of individual cells and strong desynchronization, resulting in an extremely low average amplitude (Fig. 8b). Based on the geometric illustration, LIT and LID stimuli of equal intensity induced a nonmonotonic amplitude response between CT9 and CT21 while causing a monotonic increase in amplitude response with stimulus strength at other phases (Fig. 8c). To validate these findings, we employed the normal form model to apply stimuli of identical intensities to both variables simultaneously at different phases and measured the response of the average amplitude. The simulation results are consistent with the explanation provided by the geometric illustration (Fig. 8d).

Furthermore, it is important to consider the trade-off between amplitude change and phase shift caused by a single stimulus. For example, a LIT at CT19 can advance the circadian phase but results in a decrease in amplitude (Fig. 8e, g). However, by employing a combined stimulus of LIT and LID at CT19, the circadian phase can be advanced while maintaining an improved amplitude (Fig. 8f, g). On the other hand, when both LIT and LID occur at CT11, the stimulation can shift the circadian phase without decreasing the amplitude (Fig. 8h–j).

These findings highlight the potential of combined stimulus strategies to achieve specific objectives without compromising the amplitude or phase of the circadian rhythm. In conclusion, using a combined stimulus can overcome the adverse effects from single regulation and achieve wider purpose.

## Discussion

In this study, we mainly focus on the mechanism underlying the amplitude response of an oscillator population to external stimuli. To investigate the dynamic mechanism of the amplitude response, we take the circadian clock system as an example and use a simplified normal form model to simulate amplitude responses to LIT and LID at different phases and strengths. By analyzing the responses, we can infer and illustrate the underlying mechanisms within a geometric framework, which has been previously applied to elucidate the singularity phenomenon in cyanobacterial cells^20^. Furthermore, both the stimulus timing and strength collectively determine the average amplitude after perturbation by changing individual amplitudes and the synchronization of oscillators. Based on these mechanisms, we propose a combined stimulus strategy to overcome the trade-off between amplitude change and phase shift induced by a single stimulus. This finding is particularly intriguing, as combined effects of light perturbation on both mRNA synthesis and protein degradation may well be the rule rather than the exception in the operation of the circadian clock. It should be emphasized that our 2-variable model is a stable limit cycle oscillator that does not incorporate complex biochemical reactions such as transcription, translation, and kinase regulation. Therefore, the simulation results and framework interpretation seem to offer generalized mechanisms and design principles for stimulus-response systems in limit cycle oscillators. Furthermore, the geometric relationship between oscillators after simulation and the unstable steady-state point within the stable limit cycle governs the average amplitude change, suggesting that the unstable point, rather than the stable state, is a critical factor in amplitude response. Our numerical simulation and deduction using the framework are generically applicable to a wide range of stimulus-response problems, including singularity behavior.

Although the normal form model is a simplified representation, its amplitude responses resemble the experimental data more closely than those obtained from a detailed model. In experimental observations, the phase plot of protein and mRNA concentrations approaches a cyclic pattern, resembling a circular ring. However, the phase plot of the detailed model is not a circular ring, indicating a discrepancy with the experimental observations (Supplementary Fig. 4a). On the other hand, the phase plot of the simplified normal form model is near a perfect cycle, resembling the experimental results (Supplementary Fig. 4b). Therefore, the normal form model is appropriate for elucidating the effect of light on amplitude and phase. In contrast, the detailed model, owing to its incorporation of more molecular-level information, is more appropriate to address other questions of a more molecular nature. For example, detailed molecular models not only enable the examination of the effects resulting from mutations in specific circadian clock genes but also provide a clear biological interpretation of model parameters and variables. In contrast, such interpretability is lacking in normal-form models, making detailed molecular models more suitable for comprehensive investigations. Furthermore, it is worth noting that Kronauer and coworkers have extensively studied the Van der Pol oscillator, which accurately predicts the response of the human circadian clock to light stimuli^38,39^. Notably, the phase plot of the Van der Pol oscillator closely resembles a perfect circle, consistent with the results of the normal form model. This similarity further supports the validity and relevance of the normal form model in capturing essential aspects of the circadian clock’s response to light stimuli.

In this study, we provide a comprehensive analysis of two alternative hypotheses proposed in previous studies to explain singularity behavior characterized by loss of robust circadian rhythms following exposure to a critical stimulus. In this context, the loss of oscillatory behavior in the system can be attributed to two key factors: acute desynchronization among the oscillators and a significant decrease in individual amplitudes, both of which are induced by the singularity stimulus. The critical stimulus pushes oscillators from the stable limit cycle and drives them to the vicinity of the unstable singularity points within the framework, leading to the elimination of their amplitudes. Moreover, the singularity behavior occurs in a specific time window that is determined by the stimulus mode, whether it is LIT or LID. Moreover, a previous study concentrated on a specific form of permanent amplitude suppression triggered by a critical stimulus^26^. This long-term suppression of circadian rhythms occurs via light-induced protein degradation or gene expression, wherein a stable steady state coexists with stable circadian oscillations of the limit cycle type. In the context of permanent amplitude suppression, the stable steady state plays a crucial role, contrary to the findings presented in this study. Stable steady state is visible and measurable, making them easily exploitable. On the other hand, unstable steady state is invisible and needs to be inferred, yet their significance is comparable to that of stable steady-state solutions. Singularity and amplitude response depend on the relative positioning of the post-stimulus state and the unstable steady state. Therefore, the unstable steady state serves as a core factor in these phenomena.

In our study, we observed a progressive resumption of oscillations and a return to prestimulus amplitudes within one or two cycles. The time required for this recovery to the limit cycle is determined by the relaxation parameter *ε* in our model. Oscillators with low ε exhibit a slower recovery process, requiring multiple cycles to regain amplitude, which supports the hypothesis that individual amplitude loss contributes to singularity behavior. Conversely, high *ε* values lead to an immediate resumption of amplitude after the interruption, supporting the desynchronization hypothesis for singularity. Hence, different interpretations of the singularity behavior may arise due to variations in *ε* among different species.

Notably, the amplitude response is influenced by both the strengths of external cues and the magnitude of oscillators, which has been reported in much excellent literature dealing with other circadian clock responses, including entrainment, phase shift, and jetlag^36,40^. An oscillator of low amplitude, such as a clock in an elderly, mutant mouse, tends to exhibit heightened sensitivity to the same stimulus. Thus, the geometric framework can be considered a unit cycle, and stimulus strength represents the ratio between changes in variables induced by interruption and oscillator amplitude before perturbation. This perspective allows for a comprehensive understanding of the interplay between external cues, oscillator characteristics, and the resulting amplitude response.

Our study elucidates the mechanism underlying the amplitude response, particularly focusing on the singularity behavior. Moreover, we introduce a valuable tool, the geometric framework, which facilitates the comprehension and design of stimulus-response systems. The findings of this study emphasize the effectiveness of the geometric framework as an approach to illustrate the dynamic behavior of limit cycle oscillators. With its ability to provide a comprehensive understanding of the mechanisms and principles governing amplitude responses, this framework holds significant potential for advancing the study and manipulation of complex oscillatory systems.

## Method

### Mathematical model and indices

#### Normal form model

Circadian oscillators are limit cycle-based. Therefore, we applied a limit cycle normal form model with two state variables to simulate how amplitude responds to external perturbations^29^. The differential equations for the system are:

3 $$\frac{{dx}}{{dt}}=\omega y+\varepsilon x\left({a}^{2}-{x}^{2}-{y}^{2}\right)+{L}_{{LIT}}$$

4 $$\frac{{dy}}{{dt}}=-\omega x+\varepsilon y\left({a}^{2}-{x}^{2}-{y}^{2}\right)-{L}_{{LID}}$$

As the primary focus of this study centers around light stimulation of the circadian clock, its main effect lies in activating the processes of transcription and translation for proteins. Accordingly, in this study, the symbols *x* and *y* are employed to represent the concentrations of mRNA and proteins, respectively. When exploring the influence of external stimuli on the oscillatory system, the interpretations of *x* and *y* may vary, primarily contingent upon the regulatory effects exerted by the stimuli on the variables within the system. Therefore, stimulation is implemented in the system (Eqs. (3) and (4)) by light-induced transcription (LIT) or light-induced protein degradation (LID). When light activates gene expression, *L*_*LIT*_ = 3 and *L*_*LID*_ = 0. When light triggers protein degradation, *L*_*LIT*_ = 0 and *L*_*LID*_ = 3.

The mouse SCN contains ~20,000 neurons, and the normal form model is modified to a multicellular model with multiple coupled self-sustained oscillators to simulate cell populations. The evolution equations for oscillators are written as

5 $$\frac{{{dx}}_{i}}{{dt}}={\omega }_{i}{y}_{i}+{\varepsilon x}_{i}\left({a}_{i}^{2}-{x}_{i}^{2}-{y}_{i}^{2}\right)+{L}_{{LIT}}+{k}_{c}\frac{F}{F+{K}_{f}}$$

6 $$\frac{{{dy}}_{i}}{{dt}}={\omega }_{i}{x}_{i}+{\varepsilon y}_{i}\left({a}_{i}^{2}-{x}_{i}^{2}-{y}_{i}^{2}\right)-{L}_{{LID}}$$

7 $$F=\frac{1}{N}\mathop{\sum }\limits_{i=1}^{N}{x}_{i}$$

where *N* is the total number of cellular clocks and $1\le i\le N$. ${\omega }_{i}$ and ${a}_{i}$ represent the angular velocity and the radius of the limit cycle of the *i*-th oscillator. The parameters ${\omega }_{i}$ and ${a}_{i}$ obey a Gaussian distribution (Supplementary Data 1 and 2) to simulate a group of slightly different oscillators.

The core circadian clock in mammals is typically determined by a group of cells within a brain nucleus, such as the suprachiasmatic nucleus (SCN). These cells exhibit rhythmic secretion of peptides or small molecules, which diffuse and mix, ultimately regulating the organism’s circadian clock. Therefore, we consider this mixing process as a form of averaging. The mean field *F* is the average *x* mRNA level. The values of the other parameters are set as $\varepsilon =0.0001\,{h}^{2}/n{m}^{2}$, ${k}_{c}=3{nm}/h$, and ${K}_{f}=20{nm}/h$.

When light activates gene expression, ${L}_{{LIT}}=3$ and ${L}_{{LID}}=0.$ When light triggers protein degradation, ${L}_{{LIT}}=0$ and ${L}_{{LID}}=3.$

Systems (2) and (3) produce autonomous oscillations under constant conditions, ${L}_{{LIT}}=0$ and ${L}_{{LID}}=0\,$(Supplementary Fig. 1a, b).

#### Detailed model

The core mechanism of the molecular clock is the interlocked transcription-translation feedback loop (TTFL). Thus, we modified a detailed model consisting of TTFL with 21 variables developed by Henry P. Mirsky and colleagues^35^.

The oscillatory module for circadian rhythm in individual cells is governed by equations and parameters in^35^. Supplementary Fig. 1c exhibits the sustained circadian rhythm generated by this model.

In mice, the circadian response to light pulses is mediated by CREB, a stimulus-induced transcription factor that is the target for many stimuli. In the circadian system, activated CREB increases *Per*1/2 mRNA synthesis by promoting DNA transcriptional efficiency. Accordingly, stimulation terms (8) and (9) are added to the equations of *Per*1/2 mRNA.

8 $$S\left({V}_{s1}+{V}_{{smax}1}\frac{{{Per}1}^{{n}_{s1}}}{{{Per}1}^{{n}_{s1}}+{K}_{s1}^{{n}_{s1}}}\right)$$

9 $$S\left({V}_{s2}+{V}_{{smax}2}\frac{{{Per}2}^{{n}_{s2}}}{{{Per}2}^{{n}_{s2}}+{K}_{s2}^{{n}_{s2}}}\right)$$

The mouse SCN contains ~20,000 neurons, and intercellular coupling of the cellular clock is achieved by coupling agents, including VIP, AVP, and other neurotransmitters^41^. These agents synchronize individual clock cells by changing their *Per* gene expression. As VIP is the most important neurotransmitter for intercellular coupling, we chose VIP as the synchronizing factor in the detailed model. VIP release is circadian, and transcription of *Vip* is regulated by the circadian cis-element E-box. Transcription of *Vip* mRNA and translation of VIP protein of the *j-*th oscillator is modeled as:

10 $$\begin{array}{l}\frac{{{dVip}}_{i}}{{dt}}\left({v}_{0,{vip}}+{v}_{1,{vip}}\frac{{{CLK}/{BMAL}1}_{j}^{{n}_{a1,{vip}}}}{{{KA}}_{{vip}}^{{n}_{a1,{vip}}}+{{CLK}/{BMAL}1}_{j}^{{n}_{a1,{vip}}}}\right)\left(\frac{{{KI}3}_{{vip}}^{{n}_{i3,{vip}}}}{{{KI}3}_{{vip}}^{{n}_{i3,{vip}}}+{PER}2/{{CRY}1}_{{vip}}^{{n}_{i3,{vip}}}}\right)\\\left(\frac{{{KI}4}_{{vip}}^{{n}_{i4,{vip}}}}{{{KI}4}_{{vip}}^{{n}_{i4,{vip}}}+{PER}2/{{CRY}1}_{j}^{{n}_{i3,{vip}}}}\right)-{k}_{m,{vip}}{{Vip}}_{j}\end{array}$$

11 $$\frac{{{dVIP}}_{j}}{{dt}}={t}_{{vip}}{{Vip}}_{j}-{k}_{{pvip}}{{VIP}}_{j}$$

For simplicity’s sake, we add the coupling term $\alpha ,$

12 $$\alpha ={\alpha }_{1}\frac{F}{F+{K}_{1}}$$

to the equation of *Per1* mRNA. In the coupling term, *F* is a mean-field representing the average neurotransmitter level.

13 $$F=\frac{1}{N}\mathop{\sum }\limits_{j=1}^{N}{{VIP}}_{j}$$

*N* is the total number of oscillators. The modified model produces autonomous synchronous oscillations under constant conditions, $S=0\,$(Supplementary Fig. 1d).

Then, the stimulation terms (14) and (15) are added to the equations of *Per*1/2 mRNA. ${{Per}1}_{i}$ and ${{Per}2}_{i}$ denote the concentrations of *Per1* and *Per2* mRNA of the

*i*th oscillator, respectively.

14 $$S\left({V}_{s1}+{V}_{{smax}1}\frac{{{Per}1}_{j}^{{n}_{s1}}}{{{Per}1}_{j}^{{n}_{s1}}+{K}_{s1}^{{n}_{s1}}}\right)$$

15 $$S\left({V}_{s2}+{V}_{{smax}2}\frac{{{Per}2}_{j}^{{n}_{s2}}}{{{Per}2}_{j}^{{n}_{s2}}+{K}_{s2}^{{n}_{s2}}}\right)$$

#### Parameters of the detailed model

The parameters of the *Per1* mRNA equation are specified in Supplementary Table 1, and the parameters of the external stimulation module and intercellular coupling module are specified in Supplementary Table 2.

To model the effect of molecular noise on the clock system, we assume that the period and amplitude of each oscillator are slightly different. Therefore, the parameters of the *Per1* mRNA equations obey a Gaussian distribution, which can be described as

16 $$\hat{{P}_{i,j}}={r}_{i,j}{P}_{i}$$

where $\hat{{P}_{i,j}}$ is the *i*-th parameter of the *Per1* mRNA equation for the *j*-th oscillator, ${P}_{i}$ is the *i*-th parameter of the *Per1* mRNA equation and ${r}_{i,j}$ obeys a Gaussian distribution with a mean of 1 and a standard deviation of 0.1 (Supplementary Data 3). Other parameters of the autonomous oscillation module are the same as those in^35^.

#### Indices

The amplitude response of a single oscillator to a light pulse is represented by the ratio of amplitude in the 24 hours before (*A*_*bs*_*)* and after (*A*_*as*_*)* stimulation, which is described as:

17 $${R}_{A}=\frac{{A}_{{bs}}}{{A}_{{as}}}$$

To quantify the amplitude response of the cell population to a light pulse, we defined three indices. First, we measured the average amplitude in the 24 hours before and after stimulation and denoted them as *AA*_*bs*_ and *AA*_*as*_, respectively. The average amplitude is the change between the peak and trough of the average output. Then, the ratio between *AA*_*bs*_ and *AA*_*as*_ represents the response strength, which is described as:

18 $${R}_{{AA}}=\frac{{{AA}}_{{bs}}}{{{AA}}_{{as}}}$$

Both the amplitudes of individual cells and intercellular synchronization were changed upon the light pulse. Accordingly, we employed two indices to measure the contributions of these two aspects. We used *R*_IA_ to represent the amplitude changes within individual cells, which can be described as:

19 $${R}_{{IA}}=\frac{\mathop{\sum }\limits_{j=1}^{N}\frac{{A}_{j,{as}}}{{A}_{j,{bs}}}}{N}$$

Where ${A}_{i,{bs}}$ and ${A}_{j,{as}}$ are the amplitudes of the *j*th oscillator in the 24 hours before and after the light pulse, respectively, and *N* is the total number of oscillators. *R*_IA_ = 1 when the average ratio of amplitudes remains unchanged after light pulse. In addition, changes in intercellular synchronization were measured by *R*_IS_:

20 $${R}_{{IS}}=\frac{\left|\mathop{\sum }\limits_{j=1}^{N}{e}^{{i{\theta }}_{j,{as}}}\right|}{\left|\mathop{\sum }\limits_{j=1}^{N}{e}^{{i{\theta }}_{j,{bs}}}\right|}$$

where ${\theta }_{j,{bs}}$ and ${\theta }_{j,{as}}$ are the phases of the *j*th oscillator in the 24 hours before and after the light pulse. When the synchronization levels are unchanged, *R*_IS_ = 1. Based on the calculated *R*_IA_ and *R*_IS_, we assessed the effect of the stimulus at the single-cell and cell population levels.

### Reporting summary

Further information on experimental design is available in the Nature Research Reporting Summary linked to this article.

### Supplementary information

Supplementary Information

Supplementary Data 1

Supplementary Data 2

Supplementary Data 3

reporting-summary

## Data Availability

All data generated or analyzed during this study are included in this published article and Supplementary Information.

## Code availability

All code for simulation and analysis can be found in Supplementary Information.
