# Author Correction: Amplitude response and singularity behavior of circadian clock to external stimuli

**DOI:** 10.1038/s41540-023-00328-y

**Published:** 2023-12-21

**Authors:** Tao Zhang, Yu Liu, Ling Yang

**Affiliations:** 1 Jiangsu Key Laboratory of Neuropsychiatric Diseases and Cambridge-Su Genomic Resource Center, Medical School of Soochow University, Suzhou, Jiangsu China; 2 School of Mathematical Sciences, Soochow University, Suzhou, Jiangsu China

**Keywords:** Systems biology, Computational biology and bioinformatics

Correction to: *npj Systems Biology and Applications* 10.1038/s41540-023-00300-w, published online 12 August 2023

In the Acknowledgements section, the funding source ‘We thank all the members from Yang’s Lab for helpful comments and discussions. This work was supported by grants from the National Natural Science Foundation of China [grant number 32100931 to T.Z., 11671417 to L.Y.]. The funders had no role in the study design, data collection and analysis, decision to publish, or preparation of the manuscript.’ should have read ‘We thank all the members from Yang’s Lab for helpful comments and discussions. This work was supported by grants from the National Natural Science Foundation of China[12071330 to L.Y. and 32100931 to T.Z.] and the funding from the National Key Research and Development Program of China [2018YFA0801103 to L.Y.]. The funders had no role in the study design, data collection and analysis, decision to publish, or preparation of the manuscript’. The original article has been corrected.

