# Supplementary material for: Amplitude response and singularity behavior of circadian clock to external stimuli
Source: NPJ Syst Biol Appl. 2023 Aug 12;9:39. doi: 10.1038/s41540-023-00300-w (PMC10423250; doi:10.1038/s41540-023-00300-w)
Supplement: Supplementary file 1 — Supplementary Information [file 41540_2023_300_MOESM1_ESM.pdf]

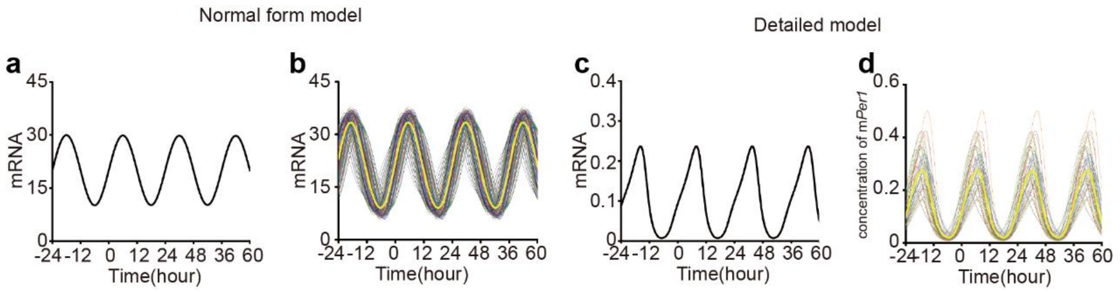

Supplementary Figure 1 Sustained oscillations generated by the normal form and detailed models. **a**-**b** Stable oscillations simulated by the normal form model for a single oscillator (**a**) and coupled oscillators (**b**). **c**-**d** Stable oscillations simulated by the detailed model for a single oscillator (**c**) and coupled oscillators (**d**). The yellow curve represents the average output.

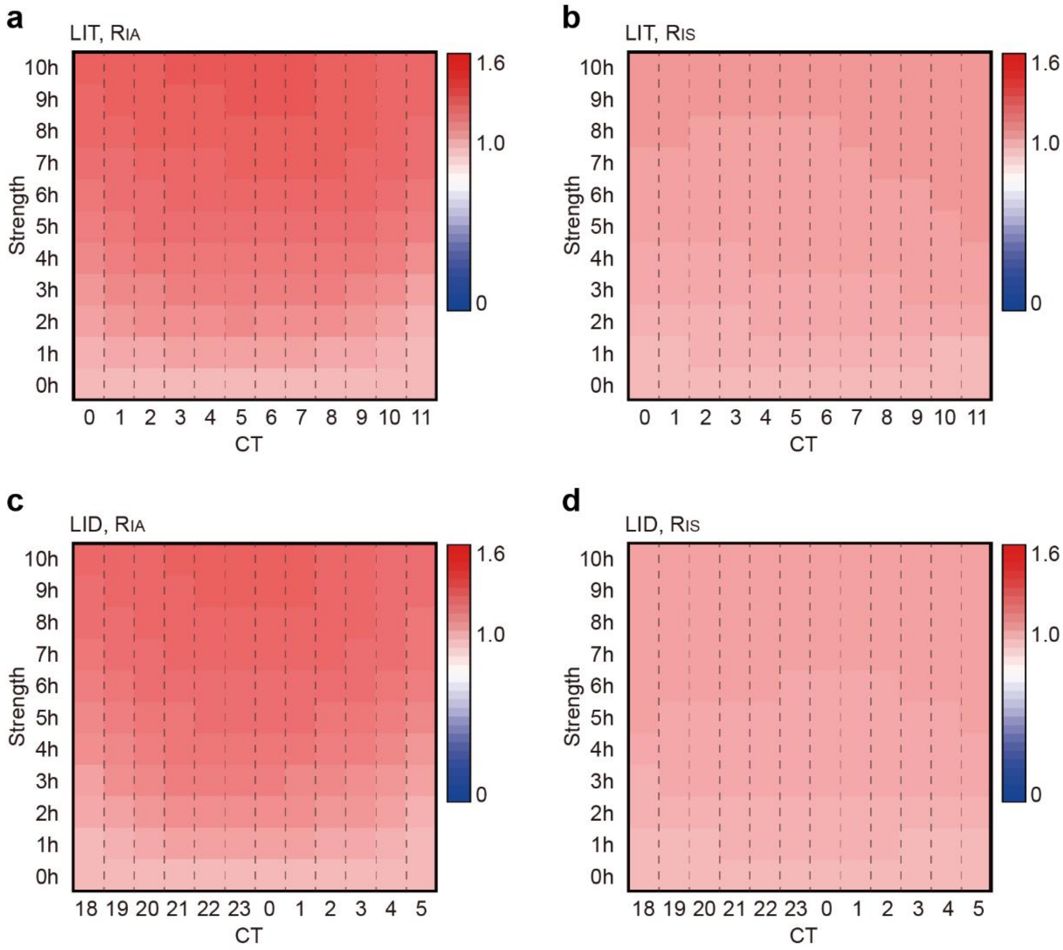

Supplementary Figure 2 Monotonic single-cell amplitude and population synchronization response. **a**-**b** Heatmaps of single-cell amplitude and population synchrony response to LIT. The values of RIA and RIS (shown in the color bar) are plotted against stimulus timing (CT0 to CT11) and strength (0 h to 10 h). **c**-**d** Heatmaps of single-cell amplitude and population synchrony response to LID. The values of RIA and RIS (shown in the color bar) are plotted against stimulus timing (CT18 to CT5) and strength (0 h to 10 h).

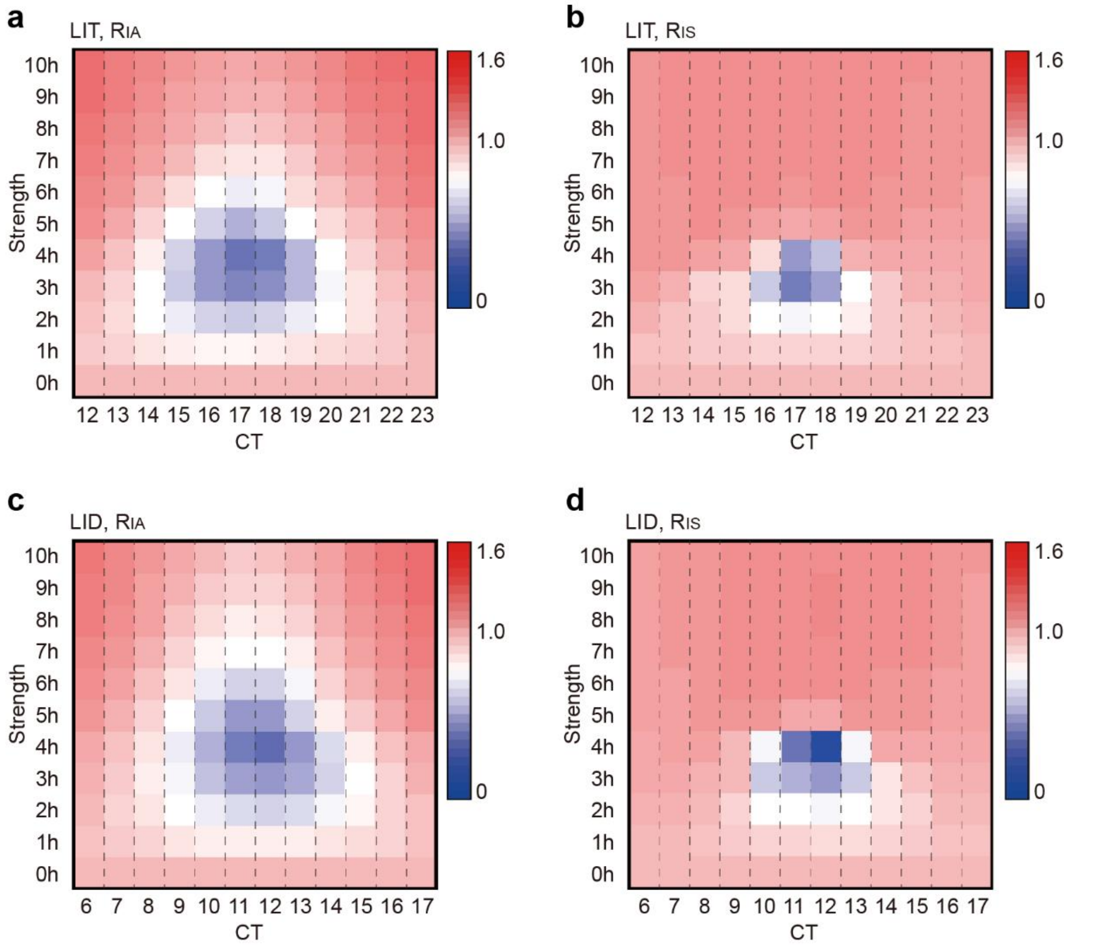

Supplementary Figure 3 Nonmonotonic single-cell amplitude and population synchrony response. **a**-**b** Heatmaps of single-cell amplitude and population synchrony response to LIT. The values of RIA and RIS (shown in the color bar) are plotted against stimulus timing (CT12 to CT23) and strength (0 h to 10 h). **c**-**d** Heatmaps of single-cell amplitude and population synchrony response to LID. The values of RIA and RIS (shown in the color bar) are plotted against stimulus timing (CT6 to CT17) and strength (0 h to 10 h).

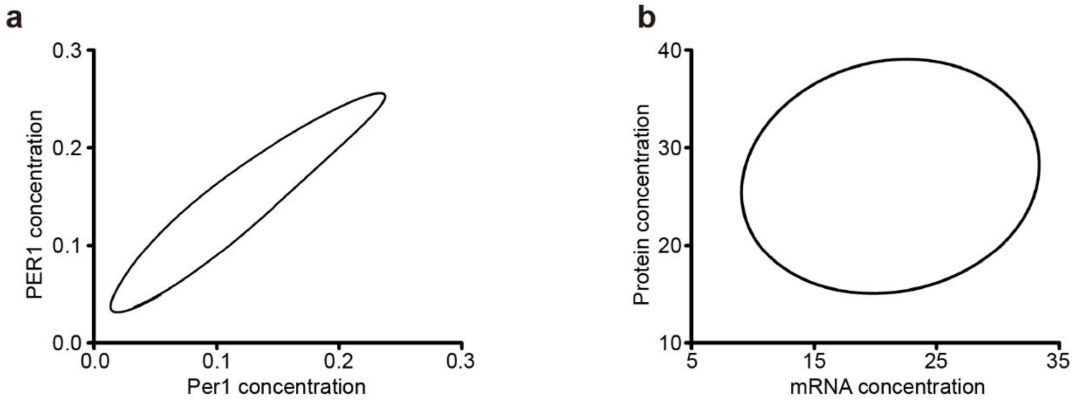

Supplementary Figure 4 The phase plot of the simplified normal form model resembles the experimental results more closely than that of the detailed model. **a** Phase plot between Per1 mRNA expression and PER1 protein simulated from the detailed model. **b** Phase plot between mRNA expression and protein simulated from the simplified normal form model.

Supplementary Table 1 Parameters of *Per1* mRNA equation

**Parameter name**	**Parameter symbol**	**Parameter value**
Basal rate of *Per1* transcription	$v_{0,per1}$	0.17 nM/h
Maximum rate of *Per1* transcription from CLK/BMAL1 activation	$v_{1,per1}$	3.84 nM/h
Hill coefficient for *Per1* transcription due to CLK/BMAL1 activation	$n_{a1,per1}$	3.28
Hill coefficient for *Per1* transcription due to PER1/CRY1 inhibition	$n_{i1,per1}$	3.93
Hill coefficient for *Per1* transcription due to PER1/CRY2 inhibition	$n_{i2,per1}$	2.61
Hill coefficient for *Per1* transcription due to PER2/CRY1 inhibition	$n_{i3,per1}$	1.66
Hill coefficient for *Per1* transcription due to PER2/CRY2 inhibition	$n_{i4,per1}$	4.85
Michaelis constant for *Per1* transcription due to CLK/BMAL1 activation	$KA_{1,per1}$	1.98 nM
Michaelis constant for *Per1* transcription due to PER1/CRY1 inhibition	$KI_{1,per1}$	1.07 nM
Michaelis constant for *Per1* transcription due to PER1/CRY2 inhibition	$KI_{2,per1}$	3.96 nM
Michaelis constant for *Per1* transcription due to PER2/CRY1 inhibition	$KI_{3,per1}$	1.68 nM
Michaelis constant for *Per1* transcription due to PER2/CRY2 inhibition	$KI_{4,per1}$	3.11 nM
Degradation rate of *Per1* mRNA	$k_{m,per1}$	2.18/h

Supplementary Table 2 Parameters of external stimulation module and intercellular coupling module

**Parameter name**	**Parameter symbol**	**Parameter value**
stimulation intensity	S	0.5 for on, 0 for off
Basal rate of *Per1* transcription by stimulation	V_{s1}	1.5 nM/h
Maximum rate of *Per1* transcription by stimulation	$V_{s\,max\,1}$	0.25 nM/h
Hill coefficient for *Per1* transcription due to stimulation	n_{s1}	6
Michaelis constant for *Per1* transcription due to stimulation	K_{s1}	3 nM/h
Basal rate of *Per2* transcription by stimulation	V_{s2}	0.0001 nM/h
Maximum rate of *Per2* transcription by stimulation	$V_{s\,max\,2}$	0.001 nM/h
Hill coefficient for *Per2* transcription due to stimulation	n_{s2}	6
Michaelis constant for *Per2* transcription due to stimulation	K_{s2}	0.3146 nM/h
Basal rate of *Vip* transcription	$v_{0,vip}$	$v_{0,per1}$
Maximum rate of *Vip* transcription from CLK/BMAL1 activation	$v_{1,vip}$	$v_{1,per1}$
Hill coefficient for *Vip* transcription due to CLK/BMAL1 activation	$n_{a1,vip}$	$n_{a1,per1}$
Hill coefficient for *Vip* transcription due to PER1/CRY1 inhibition	$n_{i1,vip}$	$n_{i1,per1}$
Hill coefficient for *Vip* transcription due to PER1/CRY2 inhibition	$n_{i2,vip}$	$n_{i2,per1}$
Hill coefficient for *Vip* transcription due to PER2/CRY1 inhibition	$n_{i3,vip}$	$n_{i3,per1}$
Hill coefficient for *Vip* transcription due to PER2/CRY2 inhibition	$n_{i4,vip}$	$n_{i4,per1}$

Michaelis constant for *Vip* transcription due to CLK/BMAL1 activation	KA_{vip}	$KA_{1,per1}$
Michaelis constant for *Vip* transcription due to PER1/CRY1 inhibition	$KI1_{vip}$	$KI_{1,per1}$
Michaelis constant for *Vip* transcription due to PER1/CRY2 inhibition	$KI2_{vip}$	$KI_{2,per1}$
Michaelis constant for *Vip* transcription due to PER2/CRY1 inhibition	$KI3_{vip}$	$KI_{3,per1}$
Michaelis constant for *Vip* transcription due to PER2/CRY2 inhibition	$KI4_{vip}$	$KI_{4,per1}$
Degradation rate of *Vip* mRNA	$k_{m,vip}$	$k_{m,per1}$

Simulation codes

Detailed model (fortran)

```
program stimuli2
implicit none
integer,parameter :: num=50
real,allocatable ::
tt(:),ave1(:),ave2(:),Per1_mRNA(:,:),Per2_mRNA(:,:),Cry1_mRNA(:,:),Cry2_mRNA
(:,:),Reverba_mRNA(:,:),Clk_mRNA(:,:),Bmal1_mRNA(:,:),Rorc_mRNA(:,:),PER1(:,
:),PER2(:,:),CRY1(:,:),CRY2(:,:),REVERBa(:,:),CLK(:,:),BMAL1(:,:),RORc(:,:),PER
1_CRY1(:,:),PER1_CRY2(:,:),PER2_CRY1(:,:),PER2_CRY2(:,:),CLK_BMAL1(:,:),
Vip_mRNA(:,:),VIP(:,:)
real,allocatable :: amp(:,:)
real
B(num,23),DD(num,23),A(4),y(num,23),D(num,23),RP(num,13),RP1(num,13),RP2(
num,13),U1(num,13),U2(num,13)
real K(num,23,4),KK(num,23)
integer i,j,s1,s2,s3,s4
real t,vmax,tau,amp0,ratio,u,F,L,mean,sigma,ph,len
real,parameter :: CP=0.3
real,parameter :: h=0.005
real,parameter :: PI=3.1415926535
real,parameter :: vmax1=0.
real,parameter :: t0=241.5
real,parameter :: tm=400
character(len=30) :: str0
character(len=30) :: str1
character(len=30) :: str2
character(len=30) :: str3
character(len=30) :: str4
```

```
character(len=30) :: str5
str0=' '
write(str3,'(f16.1)') vmax1
write(str4,'(f16.1)') CP
write(str5,'(f16.1)') t0

write(*,*) str1
write(*,*) str2
write(*,*) str3
write(*,*) str4
write(*,*) str5

A(1)=h/2.0
A(2)=h/2.0
A(3)=h
A(4)=h

s1=0
do t=0,tm,h
        s1=s1+1
end do

allocate(tt(s1))
allocate(ave1(s1))
allocate(ave2(s1))
allocate(Per1_mRNA(num,s1))
allocate(Per2_mRNA(num,s1))
allocate(Cry1_mRNA(num,s1))
allocate(Cry2_mRNA(num,s1))
allocate(Reverba_mRNA(num,s1))
```

```
allocate(Clk_mRNA(num,s1))
allocate(Bmal1_mRNA(num,s1))
allocate(Rorc_mRNA(num,s1))
allocate(PER1(num,s1))
allocate(PER2(num,s1))
allocate(CRY1(num,s1))
allocate(CRY2(num,s1))
allocate(REVERBa(num,s1))
allocate(CLK(num,s1))
allocate(BMAL1(num,s1))
allocate(RORc(num,s1))
allocate(PER1_CRY1(num,s1))
allocate(PER1_CRY2(num,s1))
allocate(PER2_CRY1(num,s1))
allocate(PER2_CRY2(num,s1))
allocate(CLK_BMAL1(num,s1))
allocate(Vip_mRNA(num,s1))
allocate(VIP(num,s1))

mean=1;
sigma=0.1
RP1(:,:)=mean+sigma*RP1(:,:);

open(unit=5,file='RPF.txt')
read(5,*) ((RP(i,j),j=1,13),i=1,num)
!!!!!!!!!!!!!!!!!!!!!!!!!!!!!!!!!!!!!!!!!!!!!!!!!!!!!!!!!!!!!!!!!!!!!!!!!!!!!!!!!!!!!!!!!!!!!!!!!!!!!!!!!!!!!!


open(unit=7,file='DD1.txt')
read(7,*) ((DD(i,j),j=1,23),i=1,num)
```

```
do ph=0,1
        do len=1,10
                write(str1,'(f16.1)') ph
                write(str2,'(f16.1)') len
                write(*,*) str1
                write(*,*) str2
!!!!!!!!!!!!!!!!!!!!!!!!!!!!!!!!!!!!!!!!!!!!!!!!!!!!!!!!!!!!!!!!!!!!!!!!!!!!!!!!!!!!!!!!!!!!!!!!!!!!!!!!
!
D(:,:)=DD(:,:)
s1=0
do t=0,tm,h
        s1=s1+1
        tt(s1)=t
        B(:,:)=D(:,:)
        F=sum(B(:,23))/num

        !light pulse
        if (t>t0+ph-len/2 .and. t<t0+ph+len/2) then
                vmax=0!vmax1
        else
                vmax=0
        end if

        do i=1,4
                call ODE1(B,y,vmax,RP,F,L,CP)
                K(:,:,i)=y(:,:)
                B(:,:)=D(:,:)+A(i)*K(:,:,i)
                F=sum(B(:,23))/num
        end do
```

```
        KK(:,:)=(K(:,:,1)+2*K(:,:,2)+2*K(:,:,3)+K(:,:,4))/6
        D(:,:)=D(:,:)+KK(:,:)*A(4)
        Per1_mRNA(:,s1)=D(:,1)
        Per2_mRNA(:,s1)=D(:,2)
        Cry1_mRNA(:,s1)=D(:,3)
        Cry2_mRNA(:,s1)=D(:,4)
        Reverba_mRNA(:,s1)=D(:,5)
        Clk_mRNA(:,s1)=D(:,6)
        Bmal1_mRNA(:,s1)=D(:,7)
        Rorc_mRNA(:,s1)=D(:,8)
        PER1(:,s1)=D(:,9)
        PER2(:,s1)=D(:,10)
        CRY1(:,s1)=D(:,11)
        CRY2(:,s1)=D(:,12)
        REVERBa(:,s1)=D(:,13)
        CLK(:,s1)=D(:,14)
        BMAL1(:,s1)=D(:,15)
        RORc(:,s1)=D(:,16)
        PER1_CRY1(:,s1)=D(:,17)
        PER1_CRY2(:,s1)=D(:,18)
        PER2_CRY1(:,s1)=D(:,19)
        PER2_CRY2(:,s1)=D(:,20)
        CLK_BMAL1(:,s1)=D(:,21)
        Vip_mRNA(:,s1)=D(:,22)
        VIP(:,s1)=D(:,23)
        ave1(s1)=sum(Per1_mRNA(:,s1))/num
        ave2(s1)=sum(PER1(:,s1))/num
    end do
```

```
s1=floor(260/h)

call Period(ave(1:s1),tt(1:s1),s1,tau)
open(unit=8,file='tau.txt')
write(8,*) tau

s1=size(tt)
open(unit=9,file='CT'//trim(adjustl( str1 ))//'d'//trim(adjustl( str2 ))//'V'//trim(adjustl( s
tr3 ))//'C'//trim(adjustl( str4 ))//'t0'//trim(adjustl( str5 ))//'P1m.txt')
do i=1,s1
      write(9,'(70f12.6)') tt(i),Per1_mRNA(:,i),ave1(i)
end do

s1=size(tt)
open(unit=10,file='CT'//trim(adjustl( str1 ))//'d'//trim(adjustl( str2 ))//'V'//trim(adjustl(
str3 ))//'C'//trim(adjustl( str4 ))//'t0'//trim(adjustl( str5 ))//'P1P.txt')
do i=1,s1
      write(10,'(70f12.6)') tt(i),PER1(:,i),ave2(i)
end do
!!!!!!!!!!!!!!!!!!!!!!!!!!!!!!!!!!!!!!!!!!!!!!!!!!!!!!!
      end do
end do




stop
end

subroutine ODE1(B,y,vmax,RP,F,L,CP)
```

```
implicit none

integer,parameter :: num=50

real B(num,23),y(num,23),x(num,23),RP(num,13)

real vmax,u,L,kl,Vl1,Vlmax1,nl1,Kl1,Vl2,Vlmax2,nl2,Kl2 !Light

real,dimension(num) ::
Per1_mRNA,Per2_mRNA,Cry1_mRNA,Cry2_mRNA,Reverba_mRNA,Clk_mRNA,
Bmal1_mRNA,Rorc_mRNA,PER1,PER2,CRY1,CRY2,REVERBa,CLK,BMAL1,RO
Rc,PER1_CRY1,PER1_CRY2,PER2_CRY1,PER2_CRY2,CLK_BMAL1,Vip_mRN
A,VIP!x(:)

real,dimension(num) ::
dPer1_mRNA,dPer2_mRNA,dCry1_mRNA,dCry2_mRNA,dReverba_mRNA,dClk_
mRNA,dBmal1_mRNA,dRorc_mRNA,dPER1,dPER2,dCRY1,dCRY2,dREVERBa,d
CLK,dBMAL1,dRORc,dPER1_CRY1,dPER1_CRY2,dPER2_CRY1,dPER2_CRY2,d
CLK_BMAL1,dVip_mRNA,dVIP !y(:)

real,dimension(num) ::
v0_per1,v1_per1,na1_per1,KA1_per1,KI1_per1,ni1_per1,KI2_per1,ni2_per1,KI3_per
1,ni3_per1,KI4_per1,ni4_per1,km_per1 !Per1_mRNA

real
v0_per2,v1_per2,na1_per2,KA1_per2,KI1_per2,ni1_per2,KI2_per2,ni2_per2,KI3_per
2,ni3_per2,KI4_per2,ni4_per2,km_per2 !Per2_mRNA

real
v0_cry1,v1_cry1,na1_cry1,KA1_cry1,v2_cry1,na2_cry1,KA2_cry1,KI1_cry1,ni1_cry
```

1,KI2_cry1,ni2_cry1,KI3_cry1,ni3_cry1,KI4_cry1,ni4_cry1,KI5_cry1,ni5_cry1,km_cry1 !Cry1_mRNA

real v0_cry2,v1_cry2,na1_cry2,KA1_cry2,v2_cry2,na2_cry2,KA2_cry2,KI1_cry2,ni1_cry2,KI2_cry2,ni2_cry2,KI3_cry2,ni3_cry2,KI4_cry2,ni4_cry2,KI5_cry2,ni5_cry2,km_cry2 !Cry2_mRNA

real v1_Reverba,na1_Reverba,KA1_Reverba,KI1_Reverba,ni1_Reverba,KI2_Reverba,ni2_Reverba,KI3_Reverba,ni3_Reverba,KI4_Reverba,ni4_Reverba,km_Reverba !Reverba_mRNA

real v0_clk,v1_clk,na1_clk,KA1_clk,KI1_clk,ni1_clk,km_clk !Clk_mRNA

real v0_Bmal1,v1_Bmal1,na1_Bmal1,KA1_Bmal1,KI1_Bmal1,ni1_Bmal1,km_Bmal1 !Bmal1_mRNA

real v0_Rorc,v1_Rorc,na1_Rorc,KA1_Rorc,v2_Rorc,na2_Rorc,KA2_Rorc,KI1_Rorc,ni1_Rorc,KI2_Rorc,ni2_Rorc,KI3_Rorc,ni3_Rorc,KI4_Rorc,ni4_Rorc,KI5_Rorc,ni5_Rorc,km_Rorc !Rorc_mRNA

real t_per1,a_per1_cry1,a_per1_cry2,d_per1_cry1,d_per1_cry2,kp_per1 !PER1

real t_per2,a_per2_cry1,a_per2_cry2,d_per2_cry1,d_per2_cry2,kp_per2 !PER2

real t_cry1,kp_cry1 !CRY1

```
real t_cry2,kp_cry2 !CRY2

real t_Reverba,kp_Reverba !REV-ERBa

real t_clk,a_clk_bmal1,d_clk_bmal1,kp_clk !CLK

real t_bmal1,kp_bmal1 !BMAL1

real t_RORc,kp_RORc !RORc

real alpha1,K1,F,CP
x(:,:)=B(:,:)

Per1_mRNA(:)=x(:,1)
Per2_mRNA(:)=x(:,2)
Cry1_mRNA(:)=x(:,3)
Cry2_mRNA(:)=x(:,4)
Reverba_mRNA(:)=x(:,5)
Clk_mRNA(:)=x(:,6)
Bmal1_mRNA(:)=x(:,7)
Rorc_mRNA(:)=x(:,8)
PER1(:)=x(:,9)
PER2(:)=x(:,10)
CRY1(:)=x(:,11)
CRY2(:)=x(:,12)
REVERBa(:)=x(:,13)
CLK(:)=x(:,14)
BMAL1(:)=x(:,15)
RORc(:)=x(:,16)
PER1_CRY1(:)=x(:,17)
```

```
PER1_CRY2(:)=x(:,18)
PER2_CRY1(:)=x(:,19)
PER2_CRY2(:)=x(:,20)
CLK_BMAL1(:)=x(:,21)
Vip_mRNA(:)=x(:,22)
VIP(:)=x(:,23)

!Per1_mRNA
v0_per1(:)=0.17*RP(:,1)
v1_per1(:)=3.84*RP(:,2)
na1_per1(:)=3.28*RP(:,3)
KA1_per1(:)=1.98*RP(:,4)
KI1_per1(:)=1.07*RP(:,5)
ni1_per1(:)=3.93*RP(:,6)
KI2_per1(:)=3.96*RP(:,7)
ni2_per1(:)=2.61*RP(:,8)
KI3_per1(:)=1.68*RP(:,9)
ni3_per1(:)=1.66*RP(:,10)
KI4_per1(:)=3.11*RP(:,11)
ni4_per1(:)=4.85*RP(:,12)
km_per1(:)=2.18*RP(:,13)

!Per2_mRNA
v0_per2=0.09
v1_per2=3.29
na1_per2=4.50
KA1_per2=1.90
KI1_per2=4.51
ni1_per2=4.45
KI2_per2=2.98
```

ni2_per2=3.70

KI3_per2=2.24

ni3_per2=4.35

KI4_per2=3.31

ni4_per2=3.67

km_per2=0.20

!Cry1_mRNA

v0_cry1=0.26

v1_cry1=2.44

v2_cry1=2.89

na1_cry1=4.91

KA1_cry1=1.46

na2_cry1=3.01

KA2_cry1=3.76

KI1_cry1=0.03

ni1_cry1=4.65

KI2_cry1=0.77

ni2_cry1=3.66

KI3_cry1=3.59

ni3_cry1=2.59

KI4_cry1=3.44

ni4_cry1=0.29

KI5_cry1=2.82

ni5_cry1=2.24

km_cry1=0.22

!Cry2_mRNA

v0_cry2=1.29

v1_cry2=2.72

v2_cry2=4.28

na1_cry2=4.39

KA1_cry2=0.69

na2_cry2=4.43

KA2_cry2=2.96

KI1_cry2=4.63

ni1_cry2=4.68

KI2_cry2=2.95

ni2_cry2=4.71

KI3_cry2=3.57

ni3_cry2=2.23

KI4_cry2=2.75

ni4_cry2=0.48

KI5_cry2=3.97

ni5_cry2=1.75

km_cry2=0.41

!Reverba_mRNA

v1_Reverba=1.03

na1_Reverba=4.40

KA1_Reverba=3.15

KI1_Reverba=3.56

ni1_Reverba=0.30

KI2_Reverba=3.62

ni2_Reverba=0.67

KI3_Reverba=4.71

ni3_Reverba=1.93

KI4_Reverba=1.23

ni4_Reverba=3.58

km_Reverba=0.60

!Clk_mRNA

v0_clk=3.98

v1_clk=3.36

na1_clk=3.50

KA1_clk=1.59

KI1_clk=0.83

ni1_clk=1.96

km_clk=3.19

!Bmal1_mRNA

v0_Bmal1=1.98

v1_Bmal1=4.12

na1_Bmal1=4.13

KA1_Bmal1=2.59

KI1_Bmal1=2.47

ni1_Bmal1=0.02

km_Bmal1=1.42

!Rorc_mRNA

v0_Rorc=0.69

v1_Rorc=3.55

na1_Rorc=1.57

KA1_Rorc=4.30

v2_Rorc=0.46

na2_Rorc=0.56

KA2_Rorc=4.89

KI1_Rorc=3.49

ni1_Rorc=2.25

KI2_Rorc=2.43

ni2_Rorc=4.73

KI3_Rorc=2.71

ni3_Rorc=3.43

KI4_Rorc=2.09

ni4_Rorc=0.84

KI5_Rorc=3.36

ni5_Rorc=4.33

km_Rorc=1.50

!PER1

t_per1=3.05

a_per1_cry1=3.57

a_per1_cry2=3.12

d_per1_cry1=1.32

d_per1_cry2=1.85

kp_per1=2.58

!PER2

t_per2=2.38

a_per2_cry1=3.81

a_per2_cry2=0.95

d_per2_cry1=1.37

d_per2_cry2=2.42

kp_per2=0.16

!CRY1

t_cry1=3.94

kp_cry1=3.03

!CRY2

t_cry2=1.69

kp_cry2=1.72

!REV-ERBa

t_Reverba=1.60

kp_Reverba=0.31

!CLOCK

t_clk=3.04

a_clk_bmal1=1.98

d_clk_bmal1=0.97

kp_clk=1.52

!BMAL1

t_bmal1=4.00

kp_bmal1=2.28

!RORc

t_RORc=1.39

kp_RORc=3.33

!light

L=vmax

Kl=0.1

VL1=1.5

Vlmax1=0.25

nl1=6

KL1=3

```
VL2=0.001

Vlmax2=0.01

nl2=6

KL2=0.3146

!coupling

alpha1=CP

K1=0.5 !1

u=1

dPer1_mRNA(:)=(v0_per1(:)+v1_per1(:)*CLK_BMAL1(:)**na1_per1(:)/(KA1_per1
(:)**na1_per1(:)+CLK_BMAL1(:)**na1_per1(:)))*(KI1_per1(:)**ni1_per1(:)/(KI1_
per1(:)**ni1_per1(:)+PER1_CRY1(:)**ni1_per1(:)))*(KI2_per1(:)**ni2_per1(:)/(KI2
_per1(:)**ni2_per1(:)+PER1_CRY2(:)**ni2_per1(:)))*(KI3_per1(:)**ni3_per1(:)/(KI
3_per1(:)**ni3_per1(:)+PER2_CRY1(:)**ni3_per1(:)))*(KI4_per1(:)**ni4_per1(:)/(
KI4_per1(:)**ni4_per1(:)+PER2_CRY2(:)**ni4_per1(:)))-km_per1(:)*Per1_mRNA(:)
+alpha1*(F/(F+K1))+L*(Vl1+Vlmax1*(Per1_mRNA/KL1)**nl1/((Per1_mRNA/KL1)
**nl1+1))

dPer2_mRNA(:)=(v0_per2+v1_per2*CLK_BMAL1(:)**na1_per2/(KA1_per2**na1_
per2+CLK_BMAL1(:)**na1_per2))*(KI1_per2**ni1_per2/(KI1_per2**ni1_per2+PE
R1_CRY1(:)**ni1_per2))*(KI2_per2**ni2_per2/(KI2_per2**ni2_per2+PER1_CRY2
(:)**ni2_per2))*(KI3_per2**ni3_per2/(KI3_per2**ni3_per2+PER2_CRY1(:)**ni3_p
er2))*(KI4_per2**ni4_per2/(KI4_per2**ni4_per2+PER2_CRY2(:)**ni4_per2))-km_
per2*Per2_mRNA(:)+Kl*L*(Vl2+Vlmax2*Per2_mRNA**nl2/(Per2_mRNA**nl2+
KL2**nl2))

dCry1_mRNA(:)=(v0_cry1+(v1_cry1*CLK_BMAL1(:)**na1_cry1/(KA1_cry1**na1
_cry1+CLK_BMAL1(:)**na1_cry1))+(v2_cry1*RORc(:)**na2_cry1/(KA2_cry1**n
```

```
a2_cry1+RORc(:)**na2_cry1)))*(KI1_cry1**ni1_cry1/(KI1_cry1**ni1_cry1+PER1_
CRY1(:)**ni1_cry1))*(KI2_cry1**ni2_cry1/(KI2_cry1**ni2_cry1+PER1_CRY2(:)*
*ni2_cry1))*(KI3_cry1**ni3_cry1/(KI3_cry1**ni3_cry1+PER2_CRY1(:)**ni3_cry1)
)*(KI4_cry1**ni4_cry1/(KI4_cry1**ni4_cry1+PER2_CRY2(:)**ni4_cry1))*(KI5_cr
y1**ni5_cry1/(KI5_cry1**ni5_cry1+REVERBa(:)**ni5_cry1))-km_cry1*Cry1_mR
NA(:)

dCry2_mRNA(:)=(v0_cry2+(v1_cry2*CLK_BMAL1(:)**na1_cry2/(KA1_cry2**na1
_cry2+CLK_BMAL1(:)**na1_cry2))+(v2_cry2*RORc(:)**na2_cry2/(KA2_cry2**n
a2_cry2+RORc(:)**na2_cry2)))*(KI1_cry2**ni1_cry2/(KI1_cry2**ni1_cry2+PER1_
CRY1(:)**ni1_cry2))*(KI2_cry2**ni2_cry2/(KI2_cry2**ni2_cry2+PER1_CRY2(:)*
*ni2_cry2))*(KI3_cry2**ni3_cry2/(KI3_cry2**ni3_cry2+PER2_CRY1(:)**ni3_cry2)
)*(KI4_cry2**ni4_cry2/(KI4_cry2**ni4_cry2+PER2_CRY2(:)**ni4_cry2))*(KI5_cr
y2**ni5_cry2/(KI5_cry2**ni5_cry2+REVERBa(:)**ni5_cry2))-km_cry2*Cry2_mR
NA(:)

dReverba_mRNA(:)=(v1_Reverba*CLK_BMAL1(:)**na1_Reverba/(KA1_Reverba*
*na1_Reverba+CLK_BMAL1(:)**na1_Reverba))*(KI1_Reverba**ni1_Reverba/(KI
1_Reverba**ni1_Reverba+PER1_CRY1(:)**ni1_Reverba))*(KI2_Reverba**ni2_Re
verba/(KI2_Reverba**ni2_Reverba+PER1_CRY2(:)**ni2_Reverba))*(KI3_Reverba
**ni3_Reverba/(KI3_Reverba**ni3_Reverba+PER2_CRY1(:)**ni3_Reverba))*(KI4
_Reverba**ni4_Reverba/(KI4_Reverba**ni4_Reverba+PER2_CRY2(:)**ni4_Reverb
a))-km_Reverba*Reverba_mRNA(:)

dClk_mRNA(:)=(v0_clk+(v1_clk*RORc**na1_clk/(RORc**na1_clk+KA1_clk**na
1_clk)))*(KI1_clk**ni1_clk/(KI1_clk**ni1_clk+REVERBa**ni1_clk))-km_clk*Clk
_mRNA

dBmal1_mRNA(:)=(v0_Bmal1+v1_Bmal1*RORc(:)**na1_Bmal1/(RORc(:)**na1_B
mal1+KA1_Bmal1**na1_Bmal1))*(KI1_Bmal1**ni1_Bmal1/(KI1_Bmal1**ni1_Bm
```

```
al1+REVERBa(:)**ni1_Bmal1))-km_Bmal1*Bmal1_mRNA(:)

dRorc_mRNA(:)=(v0_Rorc+(v1_Rorc*CLK_BMAL1(:)**na1_Rorc/(CLK_BMAL1(:
)**na1_Rorc+KA1_Rorc**na1_Rorc))+(v2_Rorc*RORc(:)**na2_Rorc/(RORc(:)**n
a2_Rorc+KA2_Rorc**na2_Rorc)))*(KI1_Rorc**ni1_Rorc/(KI1_Rorc**ni1_Rorc+P
ER1_CRY1(:)**ni1_Rorc))*(KI2_Rorc**ni2_Rorc/(KI2_Rorc**ni2_Rorc+PER1_C
RY2(:)**ni2_Rorc))*(KI3_Rorc**ni3_Rorc/(KI3_Rorc**ni3_Rorc+PER2_CRY1(:)*
*ni3_Rorc))*(KI4_Rorc**ni4_Rorc/(KI4_Rorc**ni4_Rorc+PER2_CRY2(:)**ni4_Ro
rc))*(KI5_Rorc**ni5_Rorc/(KI5_Rorc**ni5_Rorc+REVERBa(:)**ni5_Rorc))-km_R
orc*Rorc_mRNA(:)

dPER1(:)=t_per1*Per1_mRNA(:)-a_per1_cry1*PER1(:)*CRY1(:)-a_per1_cry2*PER
1(:)*CRY2(:)+d_per1_cry1*PER1_CRY1(:)+d_per1_cry2*PER1_CRY2(:)-kp_per1*
PER1(:)

dPER2(:)=t_per2*Per2_mRNA(:)-a_per2_cry1*PER2(:)*CRY1(:)-a_per2_cry2*PER
2(:)*CRY2(:)+d_per2_cry1*PER2_CRY1(:)+d_per2_cry2*PER2_CRY2(:)-kp_per2*
PER2(:)

dCRY1(:)=t_cry1*Cry1_mRNA(:)-a_per1_cry1*PER1(:)*CRY1(:)-a_per2_cry1*PE
R2(:)*CRY1(:)+d_per1_cry1*PER1_CRY1(:)+d_per2_cry1*PER2_CRY1(:)-kp_cry1
*CRY1(:)

dCRY2(:)=t_cry2*Cry2_mRNA(:)-a_per1_cry2*PER1(:)*CRY2(:)-a_per2_cry2*PE
R2(:)*CRY2(:)+d_per1_cry2*PER1_CRY2(:)+d_per2_cry2*PER2_CRY2(:)-kp_cry2
*CRY2(:)

dREVERBa(:)=t_Reverba*Reverba_mRNA(:)-kp_Reverba*REVERBa(:)

dCLK(:)=t_clk*Clk_mRNA(:)-a_clk_bmal1*CLK(:)*BMAL1(:)+d_clk_bmal1*CLK
```

_BMAL1(:)-kp_clk*CLK(:)

dBMAL1(:)=t_bmal1*Bmal1_mRNA(:)-a_clk_bmal1*CLK(:)*BMAL1(:)+d_clk_bmal1*CLK_BMAL1(:)-kp_bmal1*BMAL1(:)

dRORc(:)=t_RORc*Rorc_mRNA(:)-kp_RORc*RORc(:)

dPER1_CRY1(:)=a_per1_cry1*PER1(:)*CRY1(:)-d_per1_cry1*PER1_CRY1(:)

dPER1_CRY2(:)=a_per1_cry2*PER1(:)*CRY2(:)-d_per1_cry2*PER1_CRY2(:)

dPER2_CRY1(:)=a_per2_cry1*PER2(:)*CRY1(:)-d_per2_cry1*PER2_CRY1(:)

dPER2_CRY2(:)=a_per2_cry2*PER2(:)*CRY2(:)-d_per2_cry2*PER2_CRY2(:)

dCLK_BMAL1(:)=a_clk_bmal1*CLK(:)*BMAL1(:)-d_clk_bmal1*CLK_BMAL1(:)

dVip_mRNA(:)=(v0_per1(:)+v1_per1(:)*CLK_BMAL1(:)**na1_per1(:)/(KA1_per1(:)**na1_per1(:)+CLK_BMAL1(:)**na1_per1(:)))*(KI1_per1(:)**ni1_per1(:)/(KI1_per1(:)**ni1_per1(:)+PER1_CRY1(:)**ni1_per1(:)))*(KI2_per1(:)**ni2_per1(:)/(KI2_per1(:)**ni2_per1(:)+PER1_CRY2(:)**ni2_per1(:)))*(KI3_per1(:)**ni3_per1(:)/(KI3_per1(:)**ni3_per1(:)+PER2_CRY1(:)**ni3_per1(:)))*(KI4_per1(:)**ni4_per1(:)/(KI4_per1(:)**ni4_per1(:)+PER2_CRY2(:)**ni4_per1(:)))-km_per1(:)*Vip_mRNA(:)

dVIP(:)=t_per1*Vip_mRNA(:)-kp_per1*VIP(:)

y(:,1)=dPer1_mRNA(:)
y(:,2)=dPer2_mRNA(:)
y(:,3)=dCry1_mRNA(:)
y(:,4)=dCry2_mRNA(:)

```
y(:,5)=dReverba_mRNA(:)
y(:,6)=dClk_mRNA(:)
y(:,7)=dBmal1_mRNA(:)
y(:,8)=dRorc_mRNA(:)
y(:,9)=dPER1(:)
y(:,10)=dPER2(:)
y(:,11)=dCRY1(:)
y(:,12)=dCRY2(:)
y(:,13)=dREVERBa(:)
y(:,14)=dCLK(:)
y(:,15)=dBMAL1(:)
y(:,16)=dRORc(:)
y(:,17)=dPER1_CRY1(:)
y(:,18)=dPER1_CRY2(:)
y(:,19)=dPER2_CRY1(:)
y(:,20)=dPER2_CRY2(:)
y(:,21)=dCLK_BMAL1(:)
y(:,22)=dVip_mRNA(:)
y(:,23)=dVIP(:)

return
end subroutine


subroutine Period(timecourse,time,s,tau)
implicit none
integer s,i
real timecourse(s),time(s)
real tau,pt1,pt2,pv1,pv2

do i=2,s-1
```

```fortran
        if(timecourse(i)>timecourse(i+1) .and.timecourse(i)>=timecourse(i-1)) then
              pv1=pv2
              pv2=timecourse(i)
              pt1=pt2
              pt2=time(i)
        end if
end do

tau=pt2-pt1
return
end

!!!!!!!!!!!!!!!!!!!!!!!!!!!!!!!!!!!!!!!!!!!!!!!!!!!!!!!!!!!!!!!!!!!!!!!!!!!!!!!!!!!!!!!!!!!!!!!!!!!!!!!!!!!!!!!!!!!!
!!!!!!!!!!!!!!!!!!!!!!!!!!!!!!!!!!!!!!!!!!!!!!!!!!!!!!!!!!!!!!!!!!!!!!!!!!!!!!!!!!!!!!!!!!!!!!!!!!!!!!!!!!!!!!!!!!!!
!!!!!!!!!!!!!!!!!!!!!!!!!!!!!!!!!!!!!!!!!!!!!!
Normal form model (Fortran)
program SLCMNCpopulation
implicit none
real t,vmax,tau,F,w0,amp0,ph
integer i,j,s1,s2,s3,s4
common s1
real,parameter :: pi=3.1415926
integer,parameter :: num=100
real
A(4),B(num,2),D(num,2),K(num,2,4),KK(num,2),y(num,2),amp(num),w(num),R1(nu
m),R2(num)
real,allocatable :: p(:,:),q(:,:),tt(:),v(:),DD1(:,:),avep(:),aveq(:)
real,parameter :: t0=905
real,parameter :: tm=2000
!real,parameter :: ph=1
```

```
real,parameter :: LL=3
real,parameter :: h=0.01
character(len=30) :: str1
character(len=30) :: str2
character(len=30) :: str3

write(str1,'(f16.1)') ph
write(str2,'(f16.1)') LL

A(1)=h/2.0
A(2)=h/2.0
A(3)=h
A(4)=h



s1=0
do t=0,tm,h
    s1=s1+1
end do

allocate(p(num,s1))
allocate(q(num,s1))
allocate(avep(s1))
allocate(aveq(s1))
allocate(v(s1))
allocate(DD1(num,2))
allocate(tt(s1))

DD1(:,1)=17.50707
DD1(:,2)=28.83635
```

```
D(:,:)=DD1(:,:)
w0=pi/12
amp0=10


open(unit=3,file='Rw.txt')
read(3,*) (w(i),i=1,num)

open(unit=4,file='Ra.txt')
read(4,*) (amp(i),i=1,num)


do ph=0,23

    D(:,:)=DD1(:,:)
    write(str1,'(f16.1)') ph
    write(str2,'(f16.1)') LL

    write(*,*) 'CT'//trim(adjustl( str1 ))//'LEN'//trim(adjustl( str2 ))
    write(*,*) t0+ph
    s1=0

    do t=0,tm,h
        s1=s1+1
        tt(s1)=t

        B(:,:)=D(:,:)
        F=sum(B(:,1))/num
```

```
        if (t>t0+ph-LL/2 .and. t<t0+ph+LL/2) then
            vmax=0!3
            v(s1)=25
        else
            vmax=0
            v(s1)=10
        end if

        do i=1,4
            call ODE(B,y,vmax,F,w,amp)
            K(:,:,i)=y(:,:)
            B(:,:)=D(:,:)+A(i)*K(:,:,i)
            F=sum(B(:,1))/num
        end do

        KK(:,:)=(K(:,:,1)+2*K(:,:,2)+2*K(:,:,3)+K(:,:,4))/6
        D(:,:)=D(:,:)+KK(:,:)*A(4)
        p(:,s1)=D(:,1)
        q(:,s1)=D(:,2)
        avep(s1)=sum(p(:,s1))/num
        aveq(s1)=sum(q(:,s1))/num
    end do

    open(unit=13,file='CT'//trim(adjustl( str1 ))//'LEN'//trim(adjustl( str2 ))//'Pm.txt')
    s1=size(tt)
    do i=floor(890/h),floor(1175/h)
        write(13,'(120f12.6)') tt(i),p(:,i),avep(i),v(i)
    end do

    open(unit=15,file='CT'//trim(adjustl( str1 ))//'LEN'//trim(adjustl( str2 ))//'Qm.txt')
```

```
        s1=size(tt)
        do i=floor(890/h),floor(1175/h)
                write(15,'(120f12.6)') tt(i),q(:,i),aveq(i),v(i)
        end do

end do

call period(avep,tt,s1,tau)
open(unit=15,file='tau.txt')
write(15,*) tau

!pause

stop
end

subroutine ODE(B,y,L,F,w,amp)
implicit none
integer,parameter :: num=100
real e,L,kc,F,kf
real,parameter :: pi=3.1415926
real B(num,2),x(num,2),y(num,2),w(num),amp(num)

e=0.0001
kc=4
kf=20!20

x(:,:)=B(:,:)
x(:,:)=x(:,:)-20
y(:,1)=w(:)*x(:,2)+e*x(:,1)*(amp(:)**2-x(:,1)**2-x(:,2)**2)+L+kc*F/(F+kf)
```

```
y(:,2)=-w(:)*x(:,1)+e*x(:,2)*(amp(:)**2-x(:,1)**2-x(:,2)**2)

return
end

subroutine Period(timecourse,time,s,tau)
implicit none
integer s,i
real timecourse(s),time(s)
real tau,pt1,pt2,pv1,pv2

do i=2,s-1
      if(timecourse(i)>timecourse(i+1) .and.timecourse(i)>=timecourse(i-1)) then
            pv1=pv2
            pv2=timecourse(i)
            pt1=pt2
            pt2=time(i)
      end if
end do

tau=pt2-pt1

return
end
```
